# Meta-analysis and systematic review of the impact of different exercise intervention on emotional symptoms in patients with bipolar affective disorders

**DOI:** 10.3389/fpsyg.2025.1706043

**Published:** 2026-01-27

**Authors:** Dandan Fan, Fangquan Deng, Yin Ji, Haijun Kong, Linjie Zhang, Yebiao Fu, Hanqiao Zhang, Junting Zhang, Shiquan Deng

**Affiliations:** 1 College of Physical Education, Kashi University, Kashi, China; 2 Physical Education Department of Shandong Jiaotong University, Jinan, China; 3 College of Sport and Leisure, Chengdu Sport University, Chengdu, China; 4 College of Aerospace Physical Education, China Civil Aviation Flight Academy, Guanghan, China; 5 College of Physical Education, Guizhou Normal University, Guiyang, China; 6 College of Electronic and Information Engineering, Sichuan Electronic Machinery Vocational and Technical College, Mianyang, China

**Keywords:** bipolar affective disorder, exercise intervention, systematic review, exercise therapy, exercise prescription

## Abstract

**Objective:**

This study systematically evaluates the effects of diverse exercise and combined interventions on patients with bipolar disorder (BD), identifies optimal intervention parameters through subgroup analyses, explores dose–response relationships, and delivers evidence-based support for exercise interventions in this population.

**Methods:**

Randomized controlled trials (RCTs) examining exercise interventions for BD patients were retrieved from domestic and international databases. Literature screening and data extraction adhered to standard criteria. Study quality was assessed via the Cochrane Risk of Bias Tool 2.0. Meta-analyses were conducted in RevMan 5.4, while subgroup analyses (stratified by intervention type, duration, frequency, and age) and dose-response analyses were performed in Stata 15. The Benjamini-Hochberg method was used to apply false discovery rate (FDR) correction, controlling false positive risks in multiple comparisons (statistical significance was defined as corrected *P* < 0.05).

**Results:**

Exercise interventions significantly improved depression, anxiety, and mania symptoms and health questionnaire outcomes in BD patients (all corrected *P* < 0.05). No significant improvements were observed in quality of life questionnaire scores or systolic blood pressure (all corrected *P* > 0.05). For diastolic blood pressure, the pooled effect lacked statistical significance (SMD = −0.113, 95% CI: −0.665–0.439, *P* = 0.688), with only one individual study showing significant improvement after correction (corrected *P* = 0.0224). Subgroup analyses revealed the following: Exercise combined with psychological or mindfulness training improved all three symptoms (depression, anxiety, mania; all corrected *P* < 0.05), whereas exercise alone improved only mania (corrected *P* < 0.05). Interventions lasting ≤ 12 and >12 weeks both improved depression, anxiety, and mania (all corrected *P* < 0.05). Exercise performed ≤ 2 sessions/week improved only anxiety (corrected *P* < 0.05), while >2 sessions/week improved only depression and mania (all corrected *P* < 0.05). Single sessions lasting ≤ 60 and >60 min both improved mania (all corrected *P* < 0.05), but only sessions >60 min improved anxiety (corrected *P* < 0.05). Patients aged ≤ 40 and >40 years both derived benefits (all corrected *P* < 0.05). Dose-response analyses indicated that anxiety and depression scores were lowest with two exercise sessions per week (*P* < 0.01).

**Conclusion:**

Exercise interventions significantly improve depression, anxiety, and mania symptoms as well as health questionnaire outcomes in BD patients, with exercise combined with psychotherapy or mindfulness training producing superior effects. Improvements in diastolic blood pressure warrant cautious interpretation, as they are supported by only one study. The recommended protocol consists of exercise combined with psychotherapy or mindfulness training, with a duration of ≥12 weeks, 2–3 sessions/week, and single-session length ≤ 90 min; this protocol exerts a positive impact on patients' emotional symptoms. Future RCTs with larger samples and longer follow-up periods are needed to further validate these findings.

**Systematic review registration:**

https://www.crd.york.ac.uk/PROSPERO/view/CRD420251032877, identifier: CRD420251032877.

## Introduction

Bipolar disorder (bipolar affective disorder) is classified by the World Health Organization as the sixth leading cause of disability and death. As a highly prevalent chronic and severe mental disorder, it is recognized as an episodic mood disorder characterized by frequent functional impairments and a reduced quality of life (Belmaker, 2004; Thomson et al., 2015). It was first identified in France in 1851, and owing to the symptom-free interval between manic and depressive episodes, the disease was initially referred to as “circular insanity.” In 1854, owing to its terrifying, hopeless, and incurable nature, it was named “manic-depressive illness.” At the turn of the nineteenth century, it was renamed “manic-depressive psychosis,” and it was not until the 1980s that the term “bipolar affective disorder” became widely adopted in the field of psychiatry (Stiles et al., 2018; Angst and Sellaro, 2000).

Patients with bipolar affective disorder experience unusually intense emotional states (mood episodes), which are known for their unpredictable and cyclical nature, leading to disruptions in emotional and behavioral patterns with each episode (Angst and Sellaro, 2000). The range of episodes spans from mania (euphoria/irritability) to severe depression, which is often accompanied by psychotic symptoms and cognitive dysfunctions. Even between episodes, patients may exhibit significant functional impairments (Belmaker, 2004). Additionally, individuals with bipolar affective disorder are at increased risk for diabetes, metabolic syndrome, and cardiovascular diseases (Alsuwaidan et al., 2009; Morriss and Mohammed, 2005; Kilbourne et al., 2009). Research shows that episodes of bipolar disorder are closely related to neurobiological factors involving impairments in cellular energy regulation, immune system abnormalities, defects in neuroprotective mechanisms, and epigenetic variations. Thus, treatment and interventions must not only focus on the physiological and psychological health of patients but also aim to prevent and reverse the adverse neurobiological changes associated with bipolar disorder (Alsuwaidan et al., 2009). Moreover, traditional psychiatric medications may increase the risk of comorbid metabolic diseases such as obesity, diabetes, and dyslipidemia in patients with psychiatric disorders (Stanton and Happell, 2014). As a result, scholars suggest that exercise could serve as a significant approach for treating and intervening in bipolar affective disorder. On the one hand, exercise can induce beneficial changes in the levels of norepinephrine and its metabolic product, 3-methoxy-4-hydroxyphenylglycol (MHPG), thus helping regulate hormonal imbalances. On the other hand, exercise promotes the secretion of endogenous cannabinoids and dopamine, enhancing anti-inflammatory effects, reducing anxiety, increasing feelings of wellbeing, and optimizing neurotransmission in relevant brain regions associated with bipolar disorder, thereby improving cognitive function through reduced inflammation (Belmaker, 2004). However, some scholars have raised concerns, arguing that exercise may have a dual effect (Wright et al., 2012; Wrann et al., 2013). On the one hand, the direct mediating effect of exercise on mood may exacerbate manic symptoms; on the other hand, the impact of exercise on the levels of cytokines, such as interleukin-6, remains debatable, and the optimal parameters of exercise intervention for bipolar disorder patients have yet to be clarified (Alsuwaidan et al., 2009). On the basis of these controversies, this study hypothesizes that exercise affects the neurobiological mechanisms of mood disorders and has intensity and duration effects. Long-duration, moderate-intensity exercise significantly improved intervention outcomes. Therefore, this review aims to further clarify the role of exercise in interventions for patients with bipolar affective disorder investigate the effects of exercise on patients with bipolar disorder during manic episodes and further quantify the specific parameters of exercise intervention for bipolar disorder, providing strong cyclical evidence for clinical treatment and intervention.

## Methods

This study follows the Preferred Reporting Items for Systematic Reviews and Meta-Analyses (PRISMA 2020) guidelines for systematic review and meta-analysis and has been registered in the international PROSPERO database with registration number CRD420251032877, https://www.crd.york.ac.uk/PROSPERO/view/CRD420251032877.

### Literature search methods

The search method employed in this study combined both subject terms and free terms, with a search date ranging from database inception to March 2025. The English search terms used included “bipolar disorder,” “psychoses manic depressive,” “type 2 bipolar disorder,” “bipolar affective psychosis,” “manic depressive psychosis,” “sport,” “exercise,” “training,” “physical activity,” “yoga,” “shadow box,” “traditional sports items,” and “randomized controlled trial.” These terms were used to search databases including PubMed, Embase, Web of Science, Scopus, and the Cochrane Library. Chinese subject terms such as bipolar disorder sports or exercise or sports training and physical activity were used to search Chinese biomedical literature databases such as CNKI, Wanfang, and VIP.

### Inclusion and exclusion criteria

This review uses the PICOST framework to define its inclusion and exclusion criteria. For inclusion, studies must be randomized controlled trials (RCTs; with no restrictions on publication language); focus on participants diagnosed with bipolar disorder (BD, regardless of age or sex); assign the intervention group to regular clinical care plus a structured physical activity program (either alone, combined with psychotherapy, or combined with mindfulness training) assign the control group to regular clinical care only (with both groups maintaining their usual daily physical activity levels to avoid confounding effects); and measure primary outcomes including depressive, anxiety, and manic symptoms; scores from health-related and life-related questionnaires; systolic blood pressure (SBP); and diastolic blood pressure (DBP). Studies were excluded if they were duplicate publications, systematic reviews/meta-analyses, theoretical papers, or cross-sectional surveys; if the experimental group used non-exercise interventions (e.g., pharmacological treatments) as the core intervention; if they were non-RCTs; or if they did not focus on BD participants. Additionally, all included studies are two-arm RCTs—each with one intervention group and one control group, where both groups use independently recruited samples and receive separate interventions—with no multiarm designs (e.g., one control group paired with multiple intervention groups) or multicomponent intervention designs (e.g., studies with two or more intervention subgroups) included. This two-arm design ensures that all control groups act as “study-specific comparators” and eliminates the risk of duplicate counting, where a single control sample might otherwise be reused across multiple intervention comparisons.

### Literature screening and data extraction

The final selection of studies was independently conducted by two researchers in strict accordance with the Cochrane evaluation criteria. To ensure the comprehensiveness of the literature retrieval, relevant Chinese and English studies on BD were retrieved from multiple databases and imported into NoteExpress software for duplicate removal. After eliminating duplicate records, the two researchers first performed an initial screening based on titles and abstracts to exclude obviously ineligible studies. A comprehensive full-text review was subsequently conducted for the remaining studies to confirm whether they met the predefined inclusion criteria; any discrepancies between the two researchers were resolved through discussion or consultation with a third senior researcher. For all included studies, key information was systematically extracted, including the first author's name, publication year, type of exercise intervention (e.g., exercise alone, exercise combined with psychological therapy), intervention duration (total study period), exercise frequency (times per week), single-session length (minutes per session), and primary/secondary outcome measures. To ensure consistency and comparability of outcome data across studies, all outcome variables were standardized via validated rating scales; the scoring direction of each scale was explicitly defined, and reverse-coded items were adjusted according to the corresponding scale manuals to align with the unified scoring logic. Standardization of outcome measures: Depressive symptoms were evaluated via either the clinician-rated Hamilton Depression Rating Scale (HAMD) or the self-reported Self-Rating Depression Scale (SDS). For both scales, a higher score indicated greater severity of depressive symptoms. Anxiety Symptoms: Assessed via the clinician-rated Hamilton Anxiety Rating Scale (HAMA) or the self-reported Self-Rating Anxiety Scale (SAS). Consistent with the depressive symptom scale results, higher scores reflected more pronounced anxiety symptoms. Manic Symptoms: Measured via the Bech–Rafaelsen Mania Rating Scale (BMRS) or the Beck–Rafaelsen Mania Scale (BRMS), with higher scores representing greater severity of manic symptoms. Health Status: Evaluated via the Patient Health Questionnaire-9 (PHQ-9), which has a scoring range of 0–27. As a scale targeting depressive symptoms, higher PHQ-9 scores indicate more severe depressive manifestations. Quality of Life: Assessed via the Quality of Life Enjoyment and Satisfaction Questionnaire (Q-LES-Q), which adopts a positive scoring system—higher scores denote better quality of life. Reverse-scored items in the Q-LES-Q were converted strictly in accordance with the official scale manual to maintain consistency in the scoring direction with other positive-scoring indicators. Blood pressure: Systolic blood pressure (SBP) and diastolic blood pressure (DBP) were recorded in millimeters of mercury (mmHg), with a threshold of ≥140/90 mmHg defined as elevated blood pressure. To eliminate potential measurement bias caused by differences in measurement instruments or protocols across studies, raw blood pressure values were standardized via Z scores. To address inconsistencies in baseline levels and variability ranges of blood pressure indicators (systolic and diastolic blood pressure) across studies—despite uniform measurement units—this study employs mean difference (MD) as the effect size metric for pooled analyses. The specific approach directly calculates the mean blood pressure difference (in mmHg) between intervention and control groups for each study, then computes the standard error of the effect size using within-study standard deviations. Meta-analysis ultimately combines these values to derive the overall MD and 95% confidence interval. This method preserves blood pressure's original unit (mmHg), circumventing the double standardization problem that could arise from “calculating standardized mean difference (SMD) following Z-score normalization.” It minimizes additional heterogeneity introduced by cross-study differences in reference means and standard deviations, rendering results more intuitively tied to clinical relevance (e.g., specific mmHg changes in blood pressure) and thus easier to interpret and apply. All procedures adhere to scale manuals and clinical guidelines, ensuring data comparability and result reliability.

### Literature quality assessment

The quality of the included studies was assessed via the Cochrane risk of bias assessment tool, which evaluates seven criteria, including random sequence generation, allocation concealment, and more. In the case of disagreements, a third researcher was included to discuss and reach a consensus (Konstantynowicz et al., 2011).

### Statistical analysis

A meta-analysis of intervention effects on outcome indicators was conducted via RevMan 5.4 statistical software. The outcomes of interest—including depression, anxiety, mania, health status, quality of life, systolic blood pressure, and diastolic blood pressure—were treated as continuous variables. The results are reported as standardized mean differences (SMDs) with 95% confidence intervals (CIs). Heterogeneity was evaluated via the χ^2^ test and the *I*^2^ statistic. A random-effects model was applied when heterogeneity was high (*I*^2^ > 50%), whereas a fixed-effects model was used when heterogeneity was low (*I*^2^ ≤ 50%). In addition, Stata software was employed to conduct sensitivity analyses, Egger's test, Begg's test, and meta-regression analyses. Effect sizes across the included two-arm studies were synthesized via a random effects model (DerSimonian–Laird method). Since all studies adopted independent two-arm designs, adjustments for multiple arms or multivariate models to control for repeated counts in control groups were not needed. To reduce potential bias arising from baseline differences among control groups across studies, several strategies were implemented: baseline comparability assessment: key baseline variables from all control groups (e.g., age, sex distribution, disease duration, and baseline outcome levels) were extracted, and heterogeneity was evaluated via the *I*^2^ statistic. An *I*^2^ < 50% was interpreted as low heterogeneity. Multivariable meta-regression: Covariates, including baseline age (continuous variable), study quality score (e.g., Jadad scale, categorical variable), and control type (placebo vs. usual care, categorical variable), were entered into the regression model to examine their influence on intervention effects and adjust for potential confounding factors. Robustness checks: Sensitivity analyses were conducted to further assess the stability of the included studies. All the statistical analyses adhered to established methodological standards, thereby ensuring the reliability and validity of the findings.

### Evidence quality evaluation

The GRADE system was used to assess the evidence quality in five domains: bias risk, indirectness, inconsistency, imprecision, and publication bias. The results were categorized into four levels: very low, low, moderate, and high.

## Results

### Literature search results and basic characteristics

**Figure 1:**
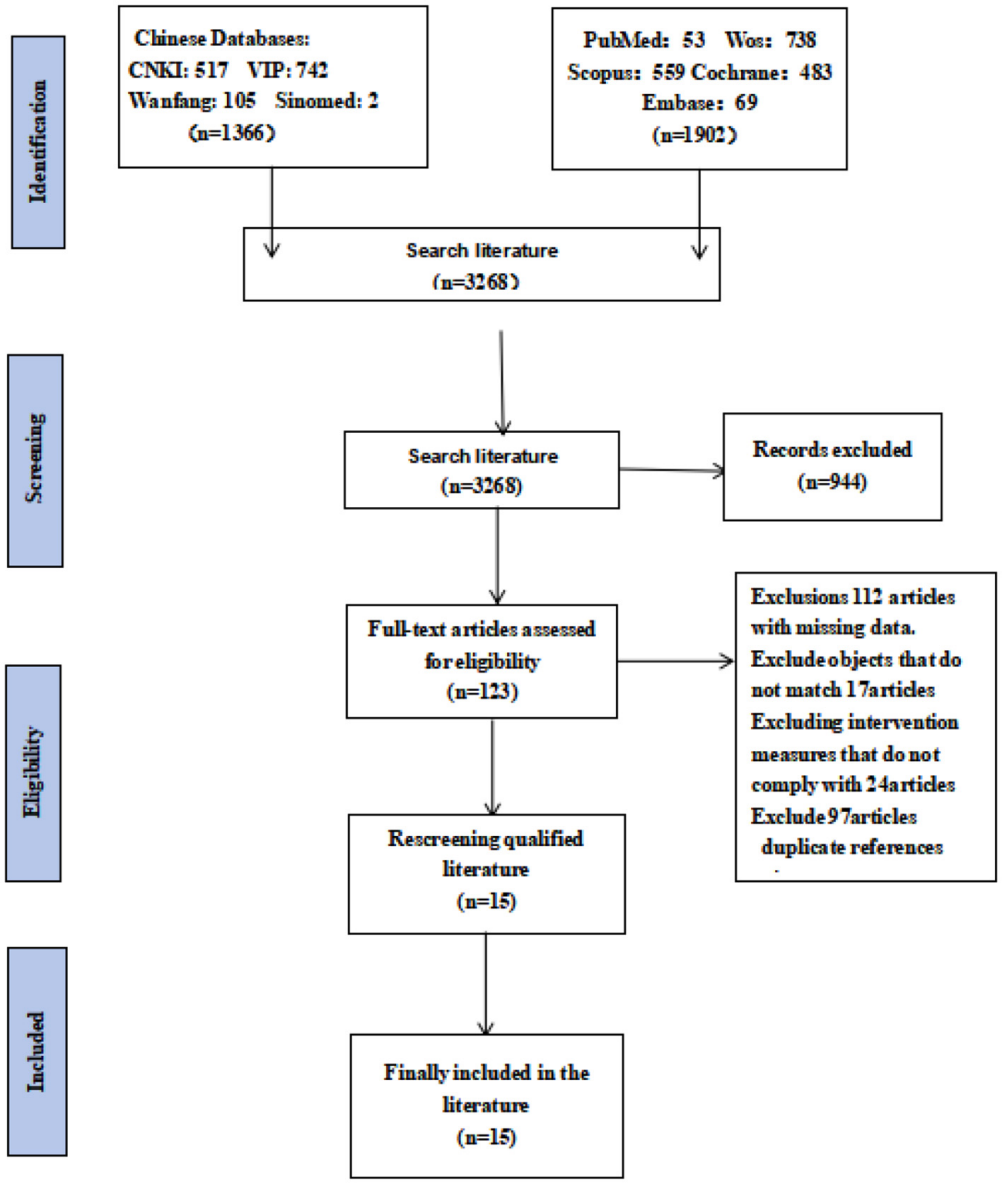
Flowchart of literature screening.

A comprehensive literature search was conducted across nine databases, including international databases (PubMed, Web of Science, Embase, Scopus, and the Cochrane Library) and Chinese databases [Chinese Biomedical Literature Database (CBM), CNKI, Wanfang Data, and VIP Database]. As presented in Figure 1, a total of 3,268 records were initially identified through thismultidatabase search. A three-step screening process was subsequently implemented to determine eligible studies: first, duplicate records were removed; second, titles and abstracts were reviewed to exclude non-relevant studies (e.g., reviews, theoretical papers); and third, full-text evaluations were performed to verify whether the remaining studies met the predefined inclusion criteria. After completing this screening process, 15 RCTs were ultimately included in the review. Characteristics of the Included Studies: All 15 included studies adopted a two-arm RCT design, with variations in control group interventions. Twelve studies used conventional treatment (i.e., routine clinical care for bipolar disorder) as the control condition, and three studies used exercise-based interventions (distinct from the exercise protocol of the experimental group) as the control condition. The experimental groups implemented three types of intervention strategies, with overlapping counts due to study design (total *n* = 15): exercise therapy alone (7 studies); combined exercise and psychological therapy (6 studies); and mindfulness-based exercise therapy (2 studies). After verifying the completeness of core exercise prescription elements—intensity, type, duration, and frequency—across included studies, this analysis identifies a significant gap in the reporting of exercise intensity parameters. Among all included studies, only one clearly reports quantitative indicators for exercise intervention intensity, defining it as 60–75% of participants' maximum heart rate (HRmax). This intensity range aligns with the “moderate-to-vigorous intensity exercise” standards recommended by the World Health Organization (WHO) and the American College of Sports Medicine (ACSM). It further specifies the HRmax measurement method (via the Bruce protocol graded exercise test), delivering critical support for the reproducibility of the exercise prescription. All other included studies fail to report any parameters related to exercise intensity. They neither utilize objective physiological indicators [e.g., percentage of HRmax, percentage of heart rate reserve (HRreserve), percentage of maximal oxygen uptake (VO_2_max)] for quantitative description nor employ subjective assessment tools [e.g., Rate of Perceived Exertion (RPE) scale, Borg scale] for qualitative explanation. Additionally, none of the studies lacking intensity data reference dynamic intensity adjustment strategies during intervention—such as increasing or decreasing intensity based on participants' exercise tolerance or changes in physiological indicators—creating critical information gaps in the core elements of the exercise prescriptions. The detailed fundamental characteristics of all included studies (e.g., sample size, intervention duration, and outcome measures) are summarized in Table 1. Baseline comparison analysis. The baseline characteristics of the control groups were analyzed to assess potential heterogeneity. Significant heterogeneity was observed in two aspects: age distribution and mania-related outcome measure scores (both *P* < 0.05). No significant heterogeneity was detected for other outcomes (e.g., depressive/anxiety symptom scores, quality of life) or disease duration (all *P* > 0.05). These results indicate that the included studies had overall good baseline comparability between the intervention and control groups. The minimal heterogeneity in age and mania-related measures was unlikely to introduce substantial bias due to baseline imbalance, as other key variables were consistent across groups. The detailed baseline data are presented in Table 2. Safety and adverse event reporting: Among the 15 included studies, 14 did not report any adverse events or safety concerns related to the intervention. The only exception was one trial, which noted that moderate-intensity aerobic exercise had no significant effect on manic symptoms (*P* > 0.05), although this finding reflects intervention efficacy rather than a safety issue.

**Table 1:** Basic characteristics of the included literature.

**First author and year**	**Number of examples**		**Intervention**		**Cyclicality**	**Frequency**	**Length of time**	**Outcome indicator**
	**EXG**	**CONG**	**EXG**	**CONG**				
Zhang et al. (2024)	45	45	Exercise+ Psychotherapy	Conventional therapy	3 weeks	^*^	^*^	1, 2, 3
Wang (2023)	40	43	Aerobic exercise	Conventional therapy	8 weeks	Twice a week	50 min	1, 2, 3, 4
Zhu et al. (2021)	74	74	Mindfulness training+ exercise therapy	Exercise therapy	8 weeks	7 times/week	90 min	1, 2, 3
Gu et al. (2021)	54	54	Exercise+ Psychotherapy	Exercise therapy	12 weeks	^*^	^*^	1, 2, 3
Duan and Li (2020)	68	60	Exercise+ Psychotherapy	Conventional therapy	36 weeks	^*^	^*^	1, 2, 3
Zhang et al. (2019)	19	19	Exercise+ Psychotherapy	Conventional therapy	20 weeks	^*^	^*^	1, 2, 3
Wang et al. (2017)	121	121	Exercise therapy	Conventional therapy	12 weeks	Twice a week	^*^	1, 5
Tong et al. (2016)	74	74	Exercise+ Psychotherapy	Exercise therapy	52 weeks	5 times/week	30–60 min	1, 2, 3, 5
Sylvia et al. (2019)	19	19	Exercise therapy	Conventional therapy	20 weeks	5 times/week	30 min	1, 3
Song et al. (2019)	40	40	Exercise+ Psychotherapy	Conventional therapy	20 weeks	5 times/week	^*^	1, 2, 3
Li et al. (2023)	55	55	Mindfulness training+ exercise therapy	Conventional therapy	8 weeks	Twice a week	30–45 min	1, 2
Soldevila Matías et al. (2024)	19	19	Exercise therapy	Conventional therapy	12 weeks	3 times/week	60 min	6, 7
Ravindran et al. (2021)	21	21	Exercise therapy	Conventional therapy	16 weeks	Twice a week	90 min	1, 5
Ciccolo et al. (2022)	24	21	Exercise therapy	Conventional therapy	12 weeks	Twice a week	60 min	1, 2
Gerber et al. (2020)	14	11	Exercise therapy	Conventional therapy	6 weeks	3 times/week	^*^	1

1. HAMD, Hamilton Depression Rating Scale; SDS, Self-Rating Depression Scale; 2. HAMA, Hamilton Anxiety Rating Scale; SAS, Self-Rating Anxiety Scale; 3. BMRS, Beck-Rafaelsen Mania Rating Scale; BRMS, Bech-Rafaelsen Mania Rating Scale; 4. PHQ-9, Patient Health Questionnaire-9; 5. QLESQ, Quality of Life Enjoyment and Satisfaction Questionnaire; 6. SBP, Systolic Blood Pressure; 7. DBP, Diastolic Blood Pressure.

*No.

**Table 2:** Baseline characteristics included in the literature.

**Outcome indicators**	**SMD**	**95% CI**	***P***	***I*^2^ %**	***P***
Age	0.003	(−0.245, 0.250)	0.983	76.6	0.00
course of disease	−0.131	(−0.269, 0.008)	0.065	39.4	0.143
Depression	−0.007	(−0.121, 0.106)	0.897	0.0	0.792
Anxiety	0.003	(−0.130, 0.136)	0.968	0.0	0.589
Mania	−0.141	(−0.285, 0.003)	0.055	55.9	0.026
Health questionnaire	0.131	(−0.134, 0.396)	0.333	0.0	0.492
Life questionnaire	−0.107	(−0.407, 0.193)	0.484	0.0	0.941
Systolic blood pressure	0.042	(−0.200, 0.285)	0.0.731	0.0	0.540
Diastolic blood pressure	−0.051	(−0.294, 0.191)	0.678	0.0	0.603

### Literature quality assessment

**Figure 2:**
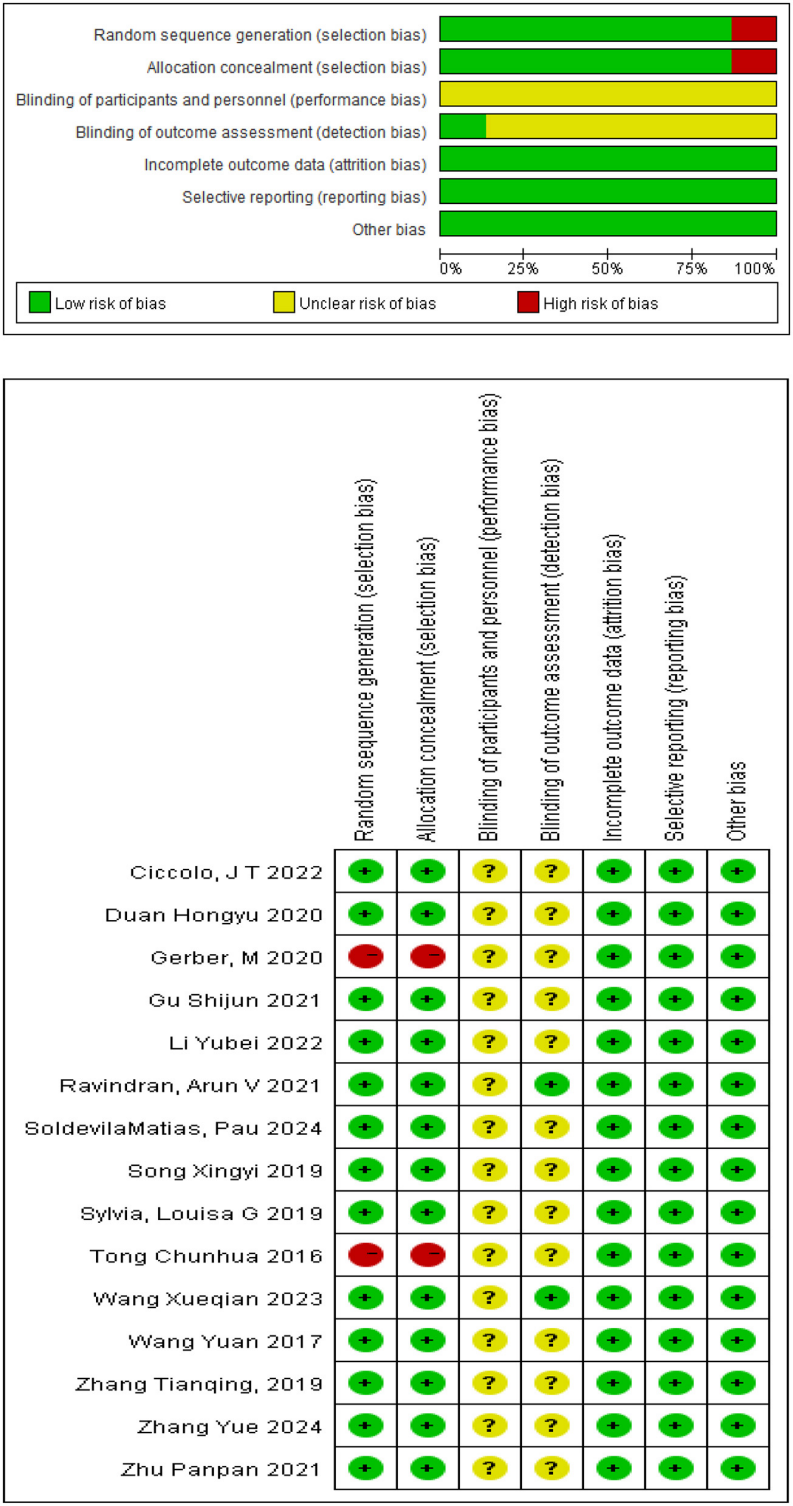
Risk assessment table for inclusion of literature bias.

The risk of bias in all included studies was evaluated via RevMan 5.4 software. The results of this assessment indicated that several studies presented a moderate level of bias risk. The primary sources of bias were as follows: randomization and allocation concealment. In this analysis, only two included studies explicitly describe specific blinding procedures (Wang, 2023; Ravindran et al., 2021). However, the original manuscript's definition of “single-blind” deviates from the internationally accepted standards outlined above—a discrepancy that could lead readers to misjudge the effectiveness of bias control. Based on the actual descriptions in the studies, the details of the blinding design are revised as follows: Both participants and outcome assessors know group assignments but remain unaware of the study's core hypothesis. This design aims to reduce subjective bias driven by “expectation bias” by concealing the research hypothesis. Specifically, it prevents participants from deliberately adjusting outcome reports (e.g., overstating or concealing symptom improvements) due to knowledge of the hypothesis, and stops outcome assessors from developing biased tendencies in scoring (e.g., assigning higher scores to groups expected to show efficacy) due to pre-conceived hypotheses. In extended bias control measures, personnel responsible for outcome assessment and statistical analysis are unaware of the specific randomization process. Outcome assessors, in particular, have no access to intervention allocation information throughout the study and only conduct objective scoring in strict adherence to standardized assessment tools (e.g., scales, measurement indicators)—a step that further reduces assessment bias. Additionally, the analysis designates independent data entry staff to manage the electronic entry of paper-based data. This ensures statistical analysts stay blinded to group assignments throughout the entire data analysis process, preventing them from introducing subjective bias into the selection of analytical strategies (e.g., outlier handling, subgroup analysis decisions) due to knowledge of group allocations. Beyond the clearly described blinding procedures noted above, no additional blinding measures are mentioned in other study processes (e.g., intervention delivery, adverse event recording). The specific workflow is presented in Figure 2.

### Meta-analysis results

#### Depression

**Figure 3:**
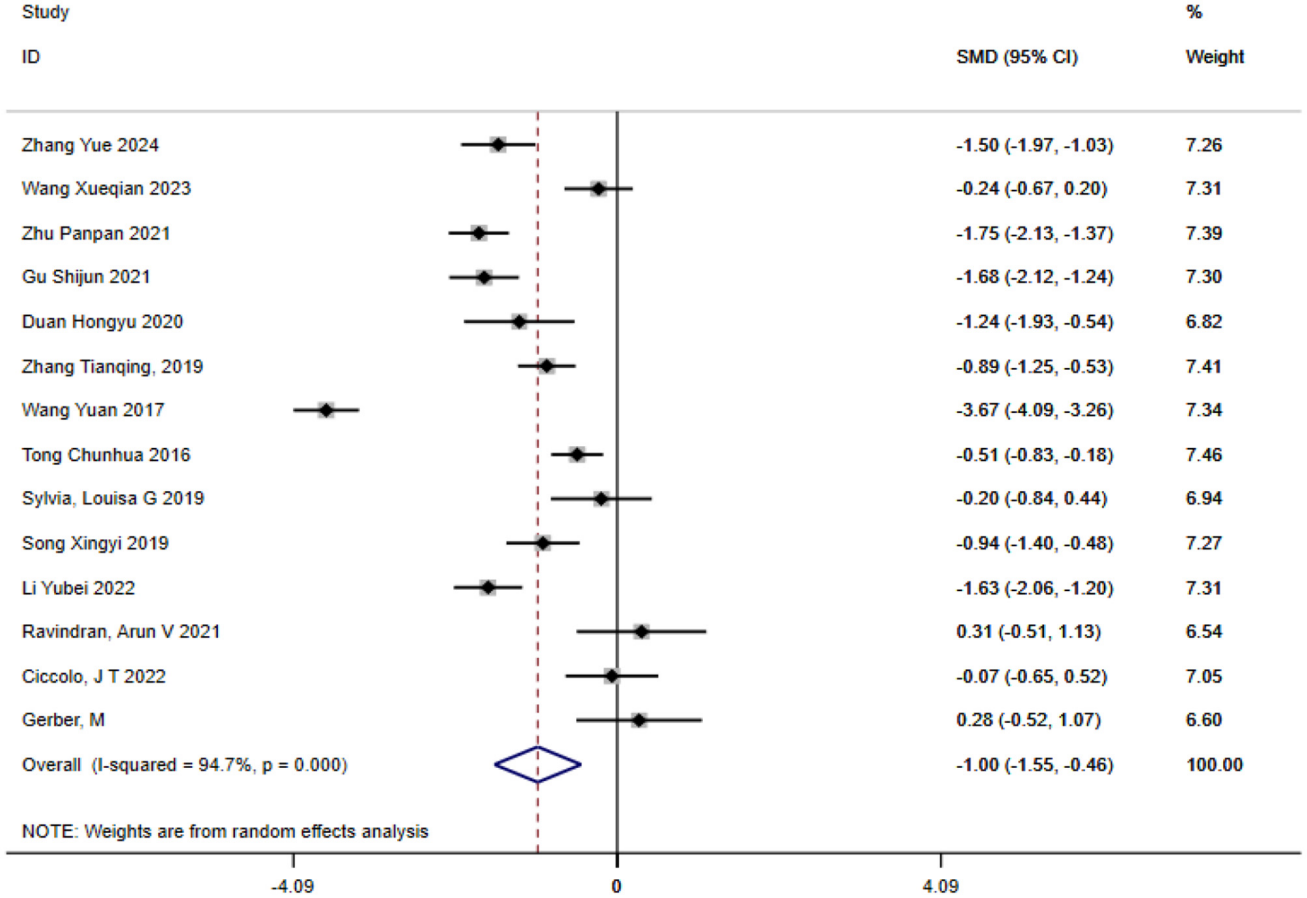
Analysis of the overall effect of depression.

A total of 14 studies were included in the analysis of exercise interventions for depression in patients with bipolar affective disorder. The overall effect test indicated significant heterogeneity among the studies (*P* = 0.00, *I*^2^ = 94.7%) thus a random effects model was used. Compared with the control group, exercise intervention had a statistically significant effect on depression in patients with bipolar affective disorder [SMD = −1.003; CI (−1.551, −0.456); *P* = 0.00], as shown in Figure 3.

#### Anxiety

**Figure 4:**
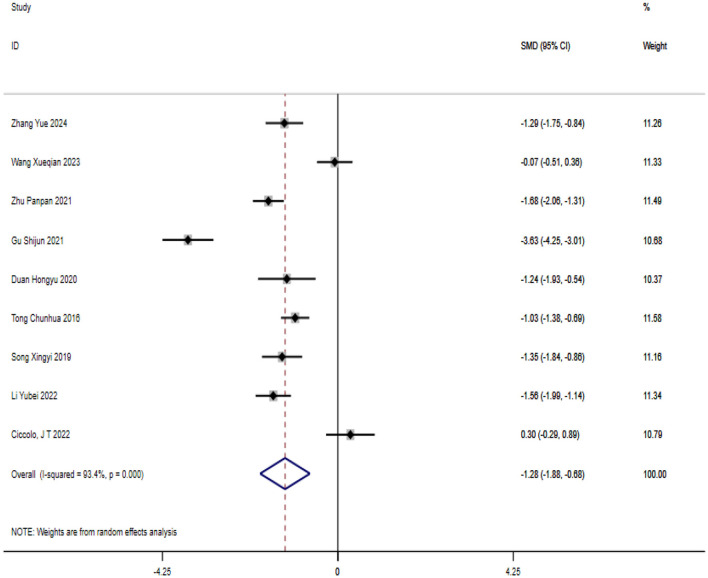
Analysis of the overall effect of anxiety.

A total of nine studies on exercise interventions for anxiety in patients with bipolar affective disorder were included. The overall effect size test indicated significant heterogeneity between the studies (*P* = 0.00, *I*^2^ = 93.4%) thus a random effects model was used. Compared with the control group, exercise intervention significantly reduced anxiety in patients with bipolar affective disorder [SMD = −1.279; CI (−1.883, −0.675); *P* = 0.00], as shown in Figure 4.

#### Mania

**Figure 5:**
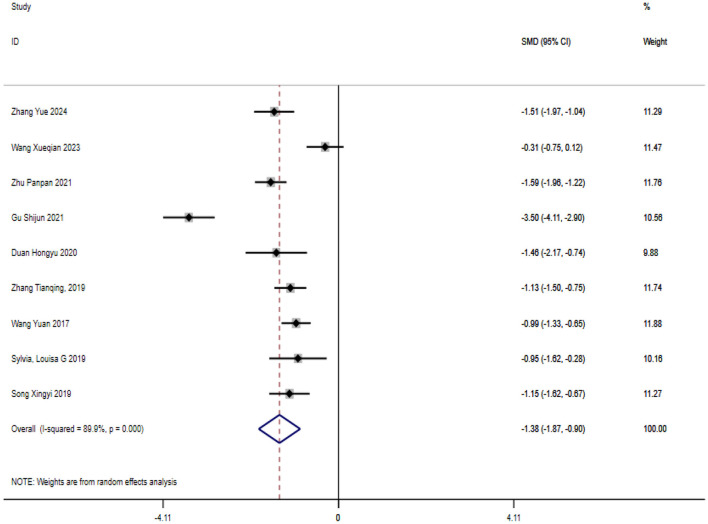
Analysis of the overall effect of mania.

A total of nine studies were included in the analysis of exercise interventions for mania in patients with bipolar affective disorder. The overall effect test indicated significant heterogeneity among the studies (*P* = 0.00, *I*^2^ = 89.9%) thus a random effects model was used. Compared with the control group, exercise intervention had a statistically significant effect on mania in patients with bipolar affective disorder [SMD = −1.382; CI (−1.867, −0.896); *P* = 0.00], as shown in Figure 5.

#### Health questionnaire

**Figure 6:**
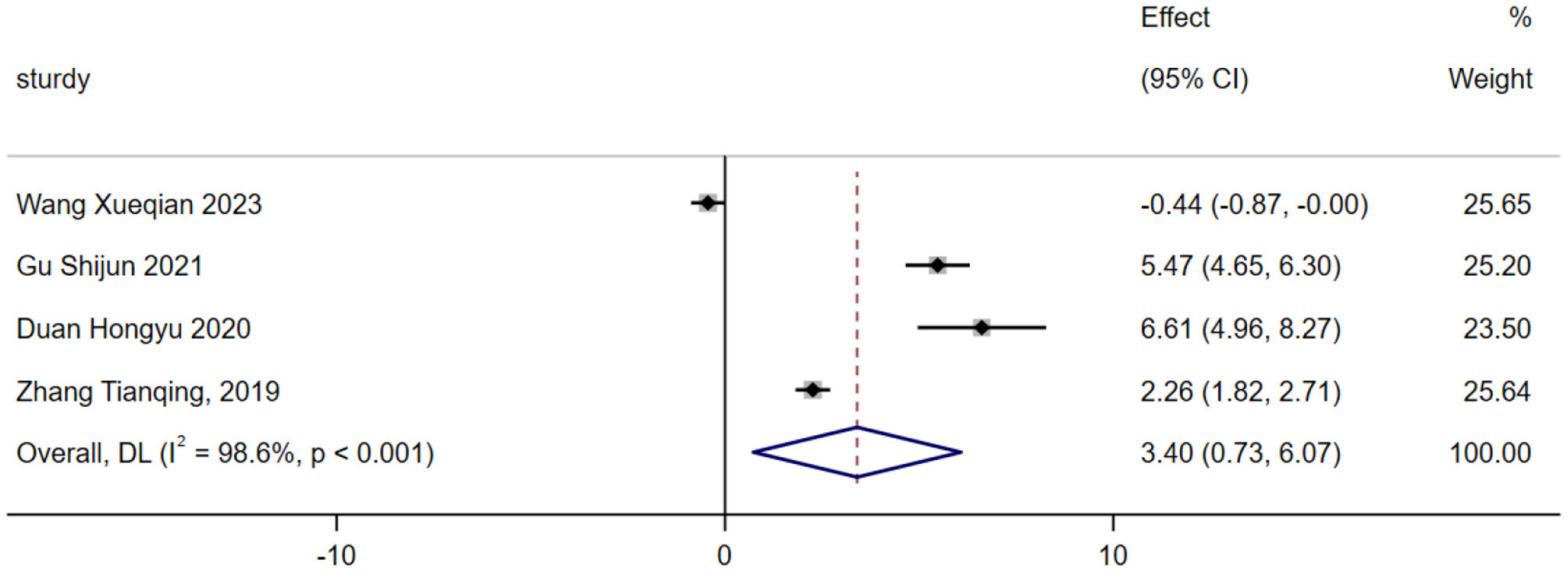
Analysis of the overall effect of the health questionnaire.

A total of four studies were included in the analysis of exercise interventions for the health questionnaire in patients with bipolar affective disorder. The overall effect test indicated significant heterogeneity among the studies (*P* = 0.00, *I*^2^ = 98.6%); thus, a random effects model was used. The analysis revealed that, compared with the control intervention, the exercise intervention had a statistically significant effect on the health questionnaire scores of patients with bipolar affective disorder [SMD = 1.607; CI (1.320, 1.894); *P* = 0.000], as shown in Figure 6.

#### Life questionnaire

**Figure 7:**
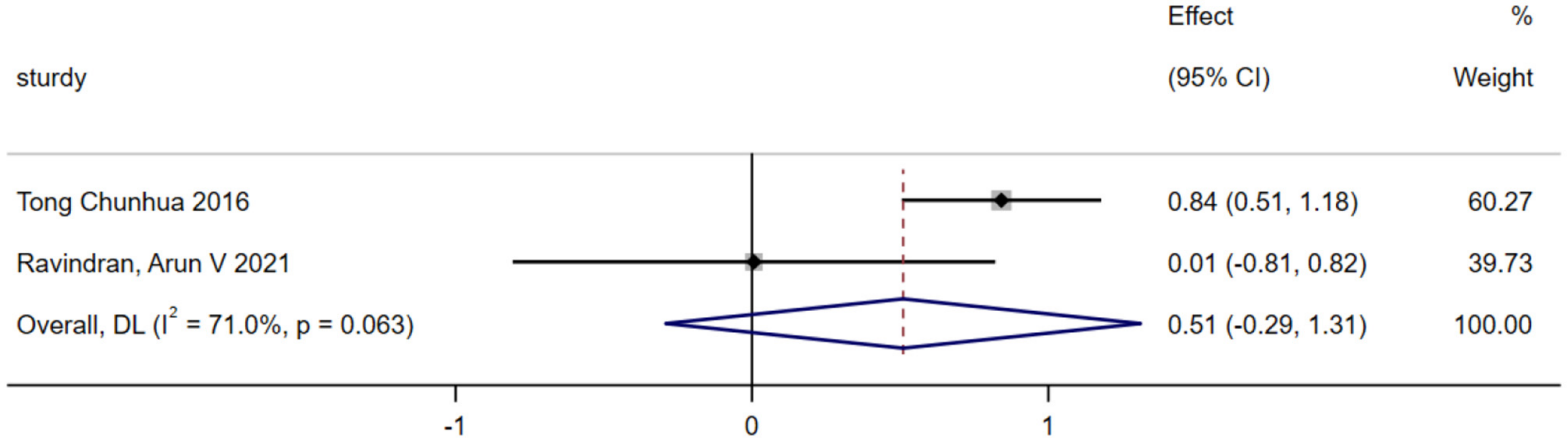
Overall utility analysis of life questionnaire.

A total of two studies were included in the analysis of exercise interventions for the life questionnaire in patients with bipolar affective disorder. The overall effect test indicated moderate heterogeneity among the studies (*P* = 0.063, *I*^2^ = 71%); thus, a random effects model was used. The analysis revealed that, compared with the control, exercise intervention had a statistically significant effect on the life questionnaire scores of patients with bipolar affective disorder [SMD = 0.510; CI (−0.291, 1.031); *P* = 0.212], as shown in Figure 7.

#### Systolic blood pressure

**Figure 8:**
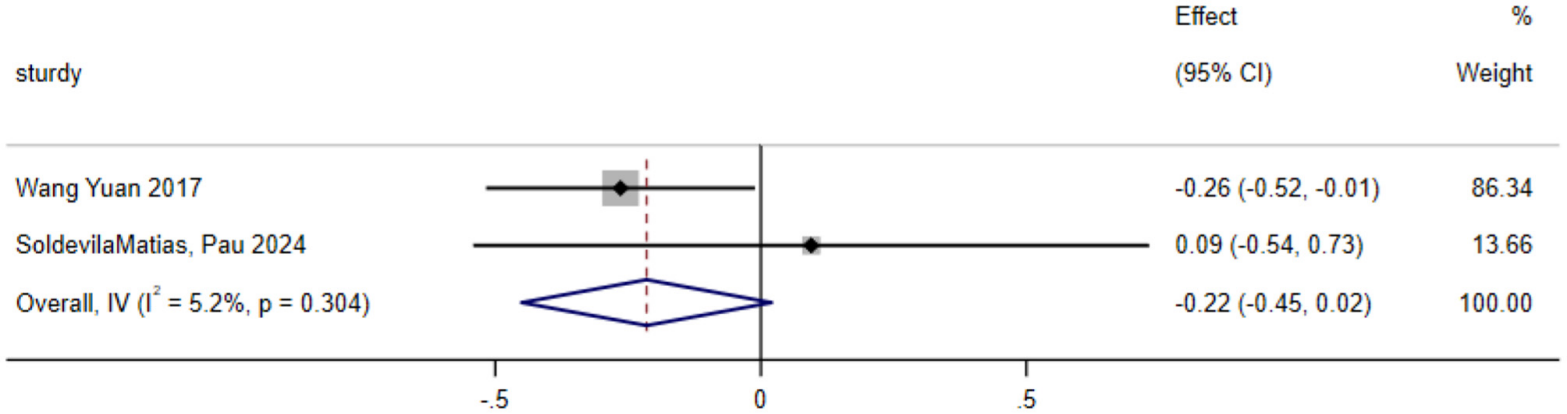
Analysis of the overall effect of systolic blood pressure.

A total of two studies were included in the analysis of exercise interventions for systolic blood pressure in patients with bipolar affective disorder. The overall effect test indicated low heterogeneity among the studies (*P* = 0.304, *I*^2^ = 5.2%); thus, a random effects model was used. The analysis revealed that, compared with the control, exercise intervention had no statistically significant effect on systolic blood pressure in patients with bipolar affective disorder [SMD = −0.215; CI (−0.450, 0.020); *P* = 0.073], as shown in Figure 8.

### Diastolic blood pressure

**Figure 9:**
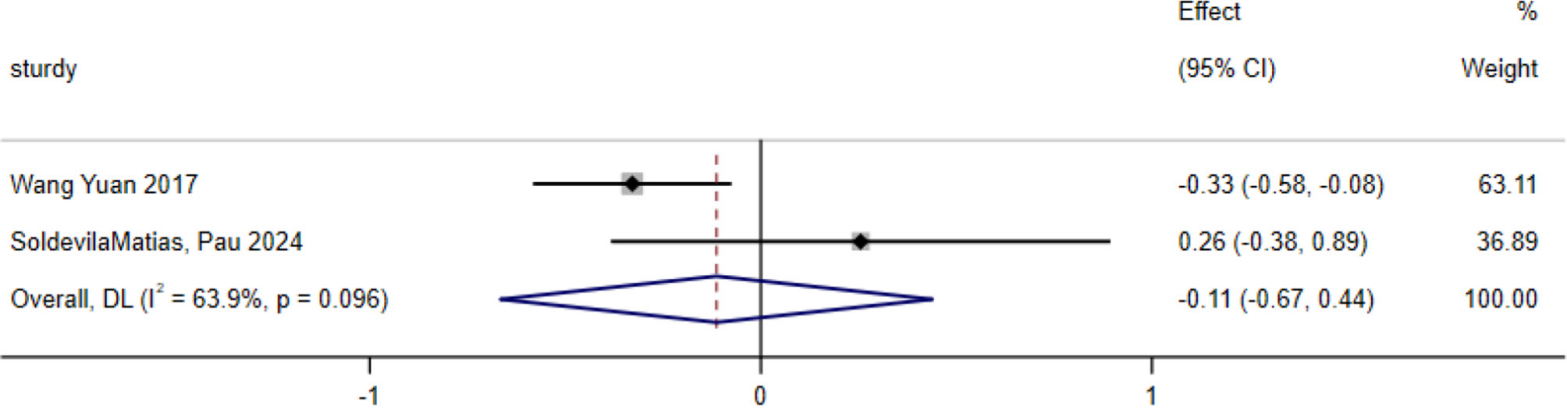
Analysis of the overall effect of diastolic blood pressure.

A total of two studies were included in the analysis of exercise interventions for diastolic blood pressure in patients with bipolar affective disorder. The overall effect test indicated moderate heterogeneity among the studies (*P* = 0.096, *I*^2^ = 63.9%); thus, a random effects model was used. The analysis revealed that, compared with the control, exercise intervention had a statistically significant effect on diastolic blood pressure in patients with bipolar affective disorder [SMD = −0.113; CI (−0.665, 0.439); *P* = 0.688], as shown in Figure 9.

### Subgroup analysis results

#### Subgroup analysis of the impact of different exercise interventions on depression in patients with bipolar affective disorders

A total of 14 studies were included in the subgroup analysis of the impact of exercise intervention on depression in patients with bipolar affective disorder. The results of the subgroup analysis are as follows: exercise combined with psychotherapy and exercise combined with mindfulness training had a significant effect on depression in patients with bipolar affective disorder (*P* < 0.05), whereas exercise alone did not have a significant effect (*P* > 0.05); intervention duration: intervention durations of ≤ 12 and >12 weeks had a significant effect on depression in patients with bipolar affective disorder (*P* < 0.05); intervention frequency: an intervention frequency of ≤ 2 times per week did not have a significant effect on depression in patients with bipolar affective disorder (*P* > 0.05), whereas an intervention frequency of >2 times per week had a significant effect on depression (*P* < 0.05); intervention duration per session: intervention sessions of ≤ 60 and >60 min did not have a significant effect on depression in patients with bipolar affective disorder (*P* > 0.05); and age: exercise intervention had a significant effect on depression in patients with bipolar disorder aged ≤ 40 and >40 years (*P* < 0.05). The specific details are shown in Table 3.

**Table 3:** Subgroup analysis of depression in bipolar disorder treated with different exercise intervention.

**Index**	**Compare types**	**95% confidence interval**	***P* value**
Intervention methods	Exercise intervention	−0.612 (−2.106, 0.883)	0.423
	Exercise combined with psychotherapy intervention	−1.105 (−1.490, −0.720)	0.000
	Exercise combined with mindfulness training intervention	−1.696 (−1.981, −1.411)	0.000
Intervention cycle	≤ 12 weeks	−1.302 (−2.145, −0.459)	0.002
	>12 weeks	−0.631 (−0.973, −0.290)	0.000
Intervention frequency	Twice a week	−1.075 (−2.579, 0.430)	0.161
	>Twice a week	−0.669 (−1.319, −0.019)	0.044
Intervention duration	≤ 60 min	−0.545 (−1.105, 0.016)	0.057
	>60 min	−0.753 (−2.765, 1.259)	0.463
Age	>40 years	−1.504 (−2.710, −0.297)	0.015
	≤ 40 years	−0.705 (−1.226, −0.185)	0.008

#### Subgroup analysis of the impact of different exercise interventions on anxiety in bipolar affective disorders

A total of nine studies were included in the subgroup analysis of the impact of exercise intervention on anxiety in patients with bipolar affective disorder. The results of the subgroup analysis are as follows: exercise combined with psychotherapy and exercise combined with mindfulness training had a significant effect on anxiety in patients with bipolar affective disorder (*P* < 0.05), whereas exercise alone did not have a significant effect (*P* > 0.05); intervention duration: intervention durations of ≤ 12 and >12 weeks had a significant effect on anxiety in patients with bipolar affective disorder (*P* < 0.05); intervention frequency: an intervention frequency of ≤ 2 times per week had a significant effect on anxiety in patients with bipolar affective disorder (*P* < 0.05), whereas an intervention frequency of >2 times per week had no significant effect on anxiety (*P* > 0.05); intervention duration per session: intervention sessions of ≤ 60 and >60 min did not have a significant effect on depression in patients with bipolar affective disorder (*P* > 0.05), whereas sessions of >60 min had a significant effect on anxiety (*P* < 0.05); and age: exercise intervention had a significant effect on anxiety in bipolar disorder patients aged ≤ 40 and>40 years (*P* < 0.05). The specific details are shown in Table 4.

**Table 4:** Subgroup analysis of different exercise Intervention for bipolar disorder anxiety.

**Index**	**Compare types**	**95% confidence interval**	***P* value**
Intervention methods	Exercise Intervention	0.059 (−0.296, 0.414)	0.744
	Exercise combined with psychotherapy intervention	−1.694 (−2.509, −0.880)	0.000
	Exercise combined with mindfulness training intervention	−1.279 (−1.883, −0.675)	0.000
Intervention cycle	≤ 12 weeks	−1.271 (−2.426, −0.115)	0.031
	>12 weeks	−1.276 (−1.530, −1.021)	0.000
Intervention frequency	Twice a week	−0.818 (−1.532, −0.104)	0.025
	>Twice a week	−1.004 (−2.086, 0.078)	0.069
Intervention duration	≤ 60 min	−0.610 (−1.388, 0.167)	0.124
	>60 min	−1.684 (−2.059, −1.308)	0.000
Age	>40 years	−0.956 (−1.651, −0.261)	0.007
	≤ 40 years	−1.271 (−1.951, −0.590)	0.024

#### Subgroup analysis of the impact of different exercise interventions on mania in bipolar affective disorders

A total of nine studies were included in the subgroup analysis of the impact of exercise intervention on mania in patients with bipolar affective disorder. The results of the subgroup analysis are as follows: intervention type: exercise alone, exercise combined with psychotherapy, and exercise combined with mindfulness training had a significant effect on mania in patients with bipolar affective disorder (*P* < 0.05); intervention duration: intervention durations of ≤ 12 and >12 weeks had a significant effect on mania in patients with bipolar affective disorder (*P* < 0.05); intervention frequency: an intervention frequency of ≤ 2 times per week did not have a significant effect on mania in patients with bipolar affective disorder (*P* > 0.05), whereas an intervention frequency of >2 times per week had a significant effect on mania (*P* < 0.05); intervention duration per session: intervention durations of ≤ 60 and >60 min both had a significant effect on mania in patients with bipolar affective disorder (*P* < 0.05); and age: exercise intervention had a significant effect on manic episodes in bipolar disorder patients aged ≤ 40 and >40 years (*P* < 0.01). The specific details are shown in Table 5.

**Table 5:** Subgroup analysis of bipolar disorder mania with different exercise intervention.

**Index**	**Compare types**	**95% confidence interval**	***P* value**
Intervention methods	Exercise intervention	−0.741 (−1.211, −0.271)	0.002
	Exercise combined with psychotherapy intervention	−1.733 (−2.515, −0.952)	0.000
	Exercise combined with mindfulness training intervention	−1.585 (−1.955, −1.215)	0.000
Intervention cycle	≤ 12 weeks	−1.711 (−2.825, −0.579)	0.003
	>12 weeks	−1.093 (−1.296, −0.890)	0.000
Intervention frequency	Twice a week	−0.313 (−0.747, 0.120)	0.156
	>Twice a week	−1.198 (−1.514, −0.882)	0.000
Intervention duration	≤ 60 min	−0.741 (−1.211, −0.271)	0.002
	>60 min	−1.585 (−1.955, −1.215)	0.000
Age	>40 years	−1.347 (−1.741, −0.953)	0.000
	≤ 40 years	−1.460 (−2.470, −0.449)	0.005

### Dose response meta-analysis

**Figure 10:**
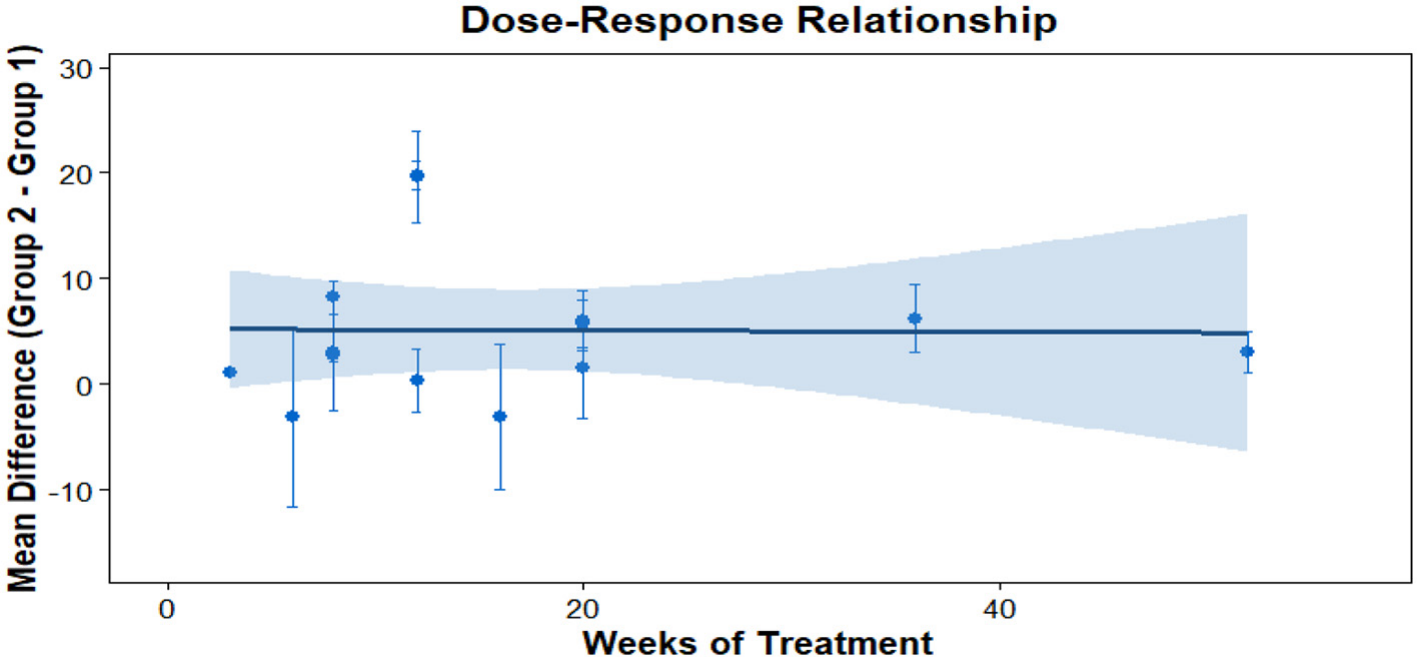
The effect of different intervention cycles on depression in patients with bipolar disorder.

**Figure 11:**
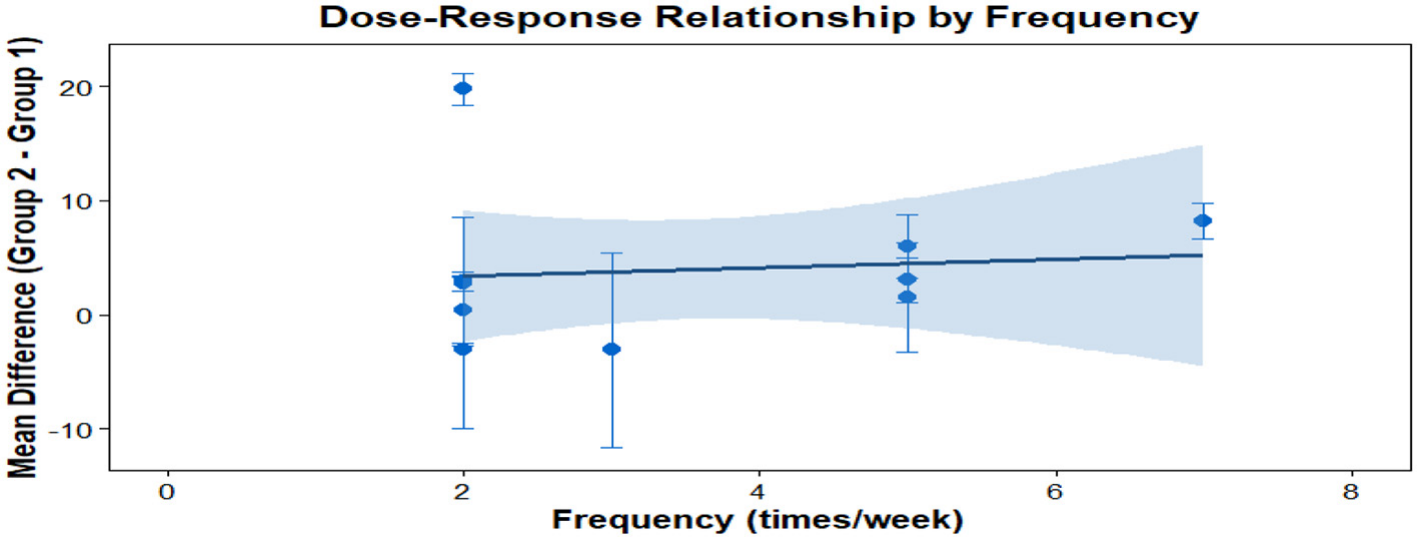
The effect of different intervention frequencies on depression in patients with bipolar disorder.

**Figure 12:**
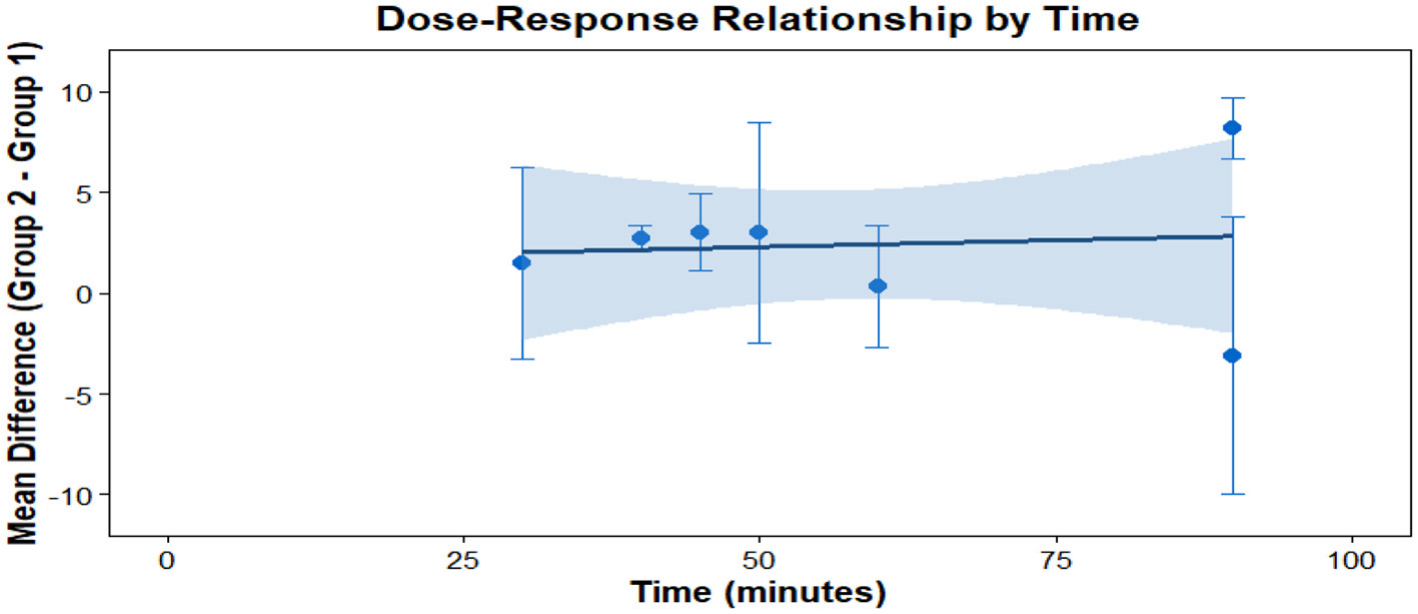
The effect of different intervention durations on depression in patients with bipolar disorder.

**Figure 13:**
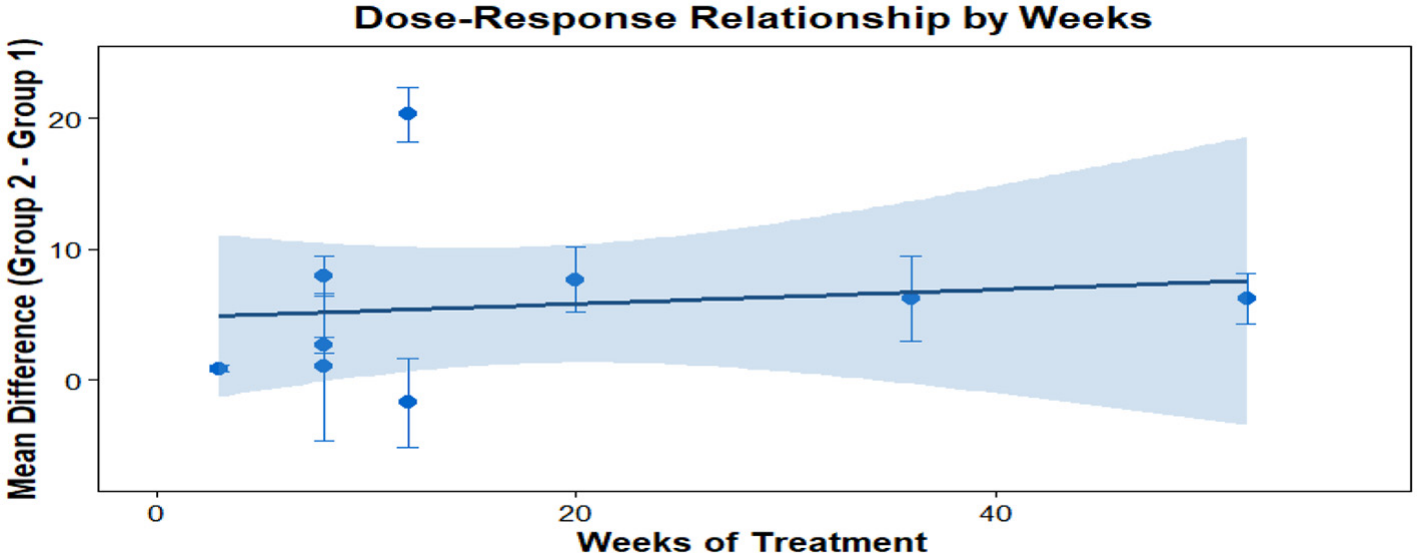
The effect of different intervention cycles on anxiety in patients with bipolar disorder.

**Figure 14:**
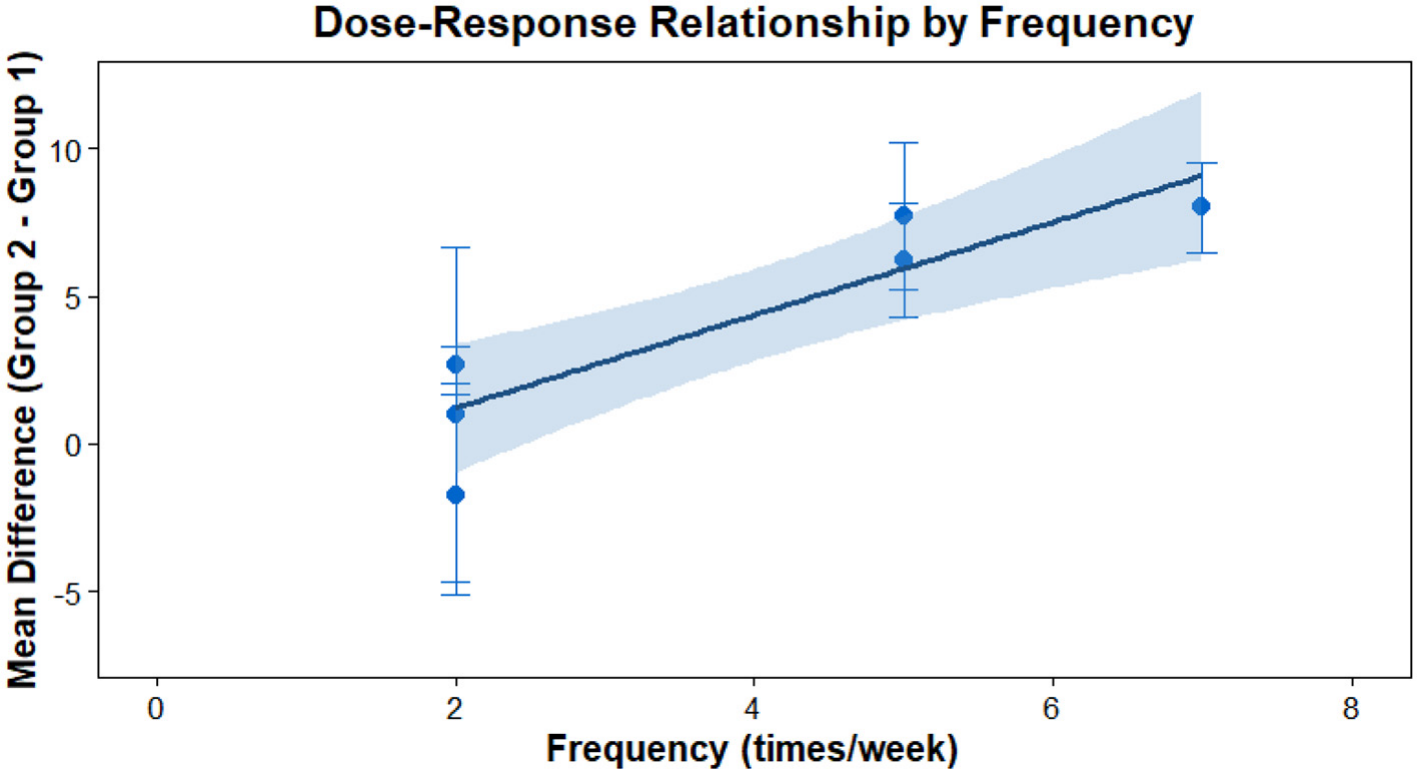
The effect of different intervention frequencies on anxiety in patients with bipolar disorder.

**Figure 15:**
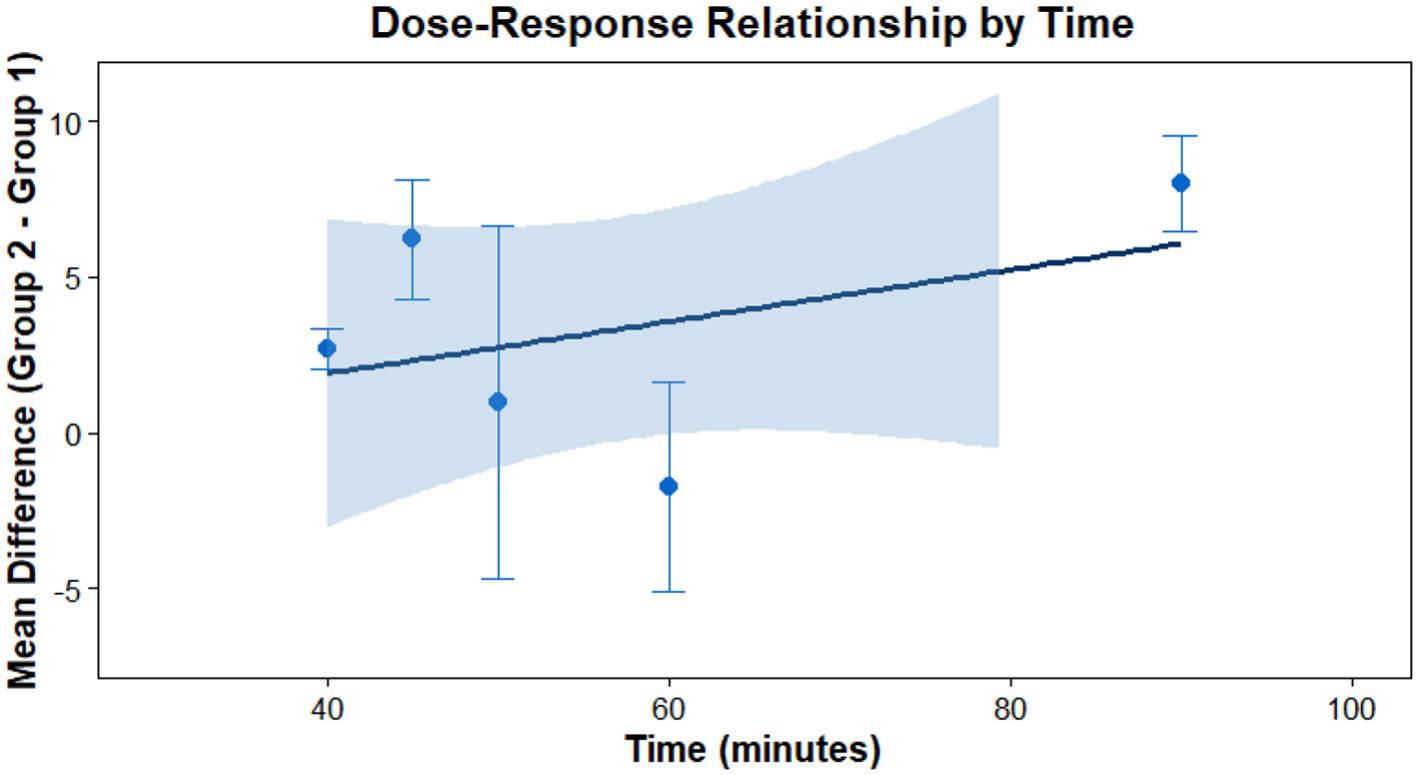
The effect of different intervention durations on anxiety in patients with bipolar disorder.

**Figure 16:**
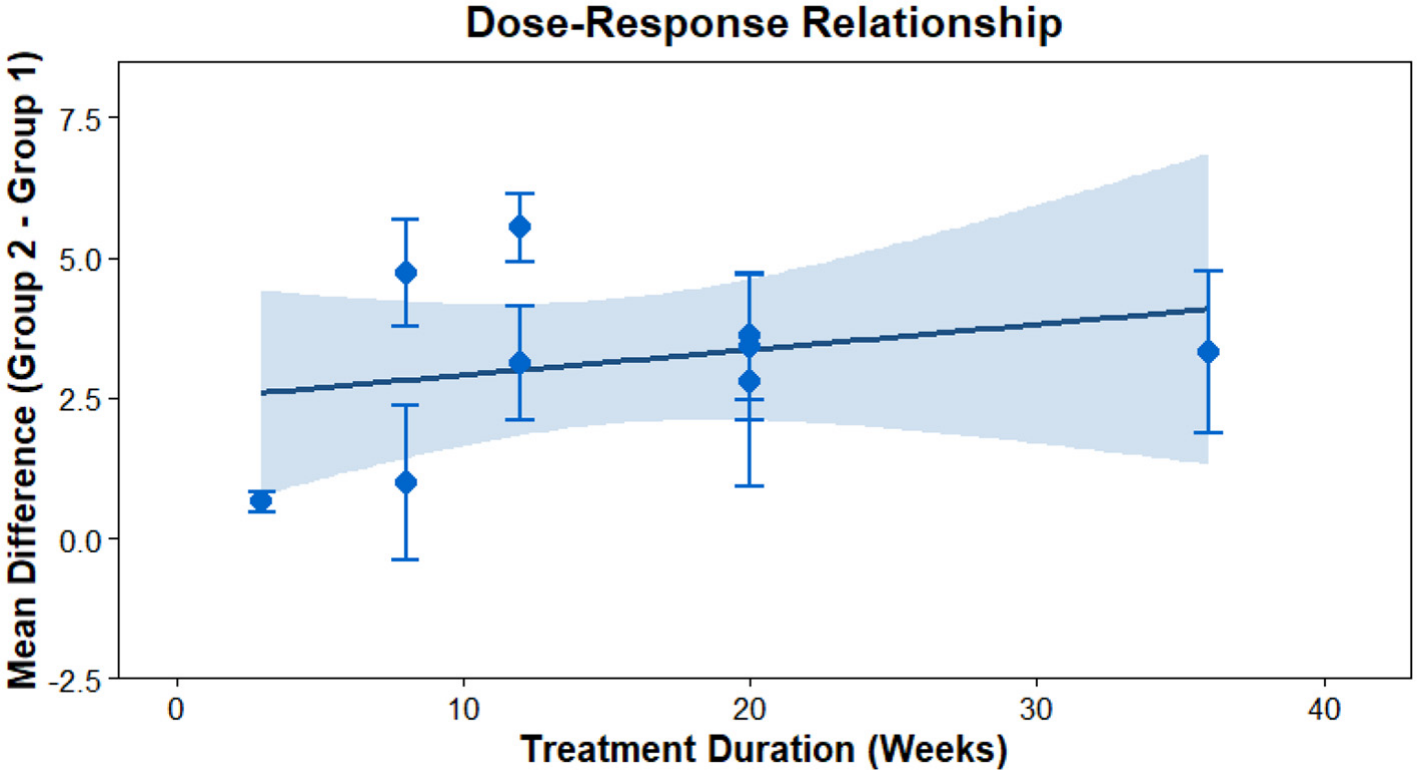
The effect of different intervention cycles on mania in patients with bipolar disorder.

**Figure 17:**
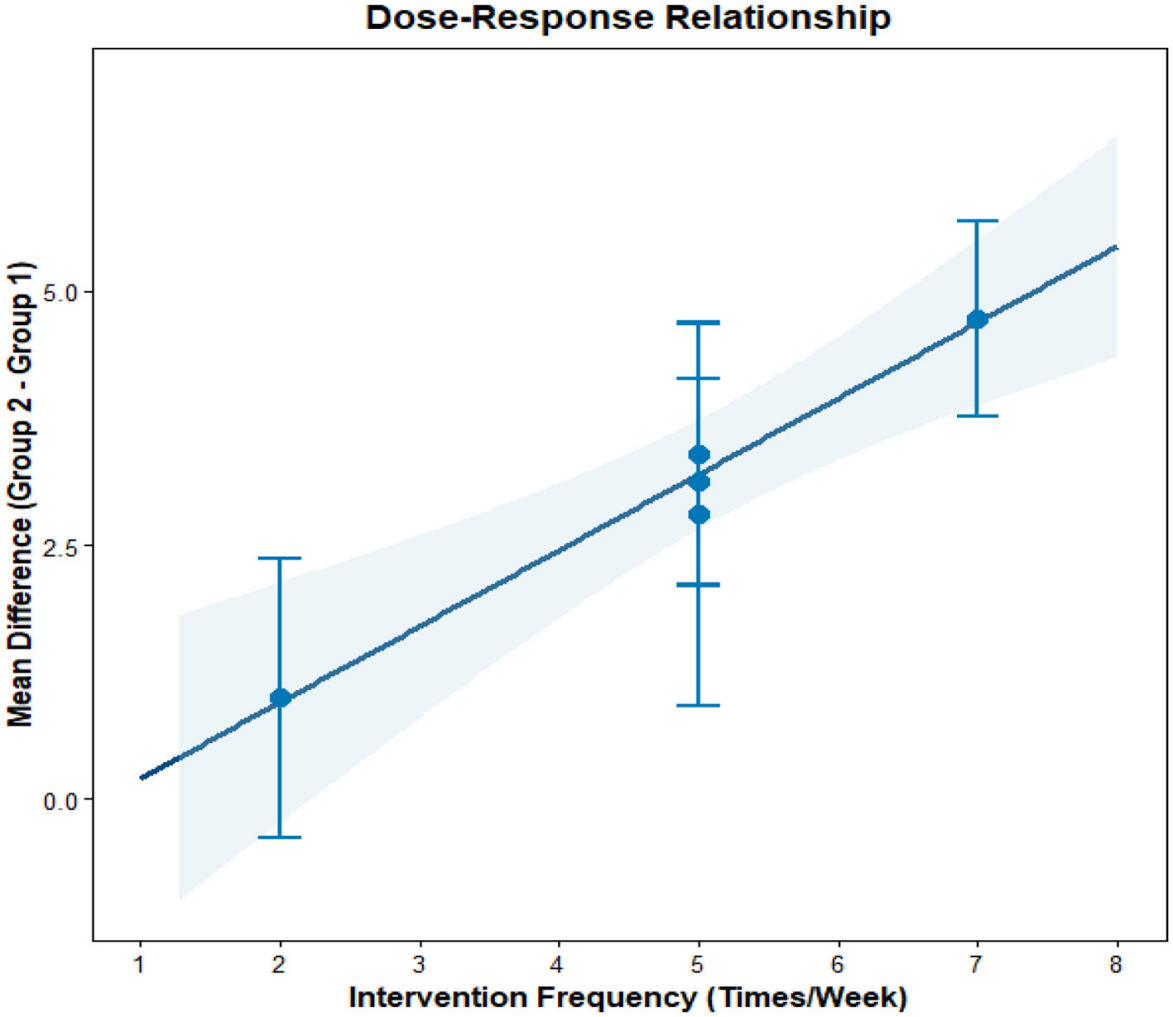
The effect of different intervention frequencies on mania in patients with bipolar disorder.

**Figure 18:**
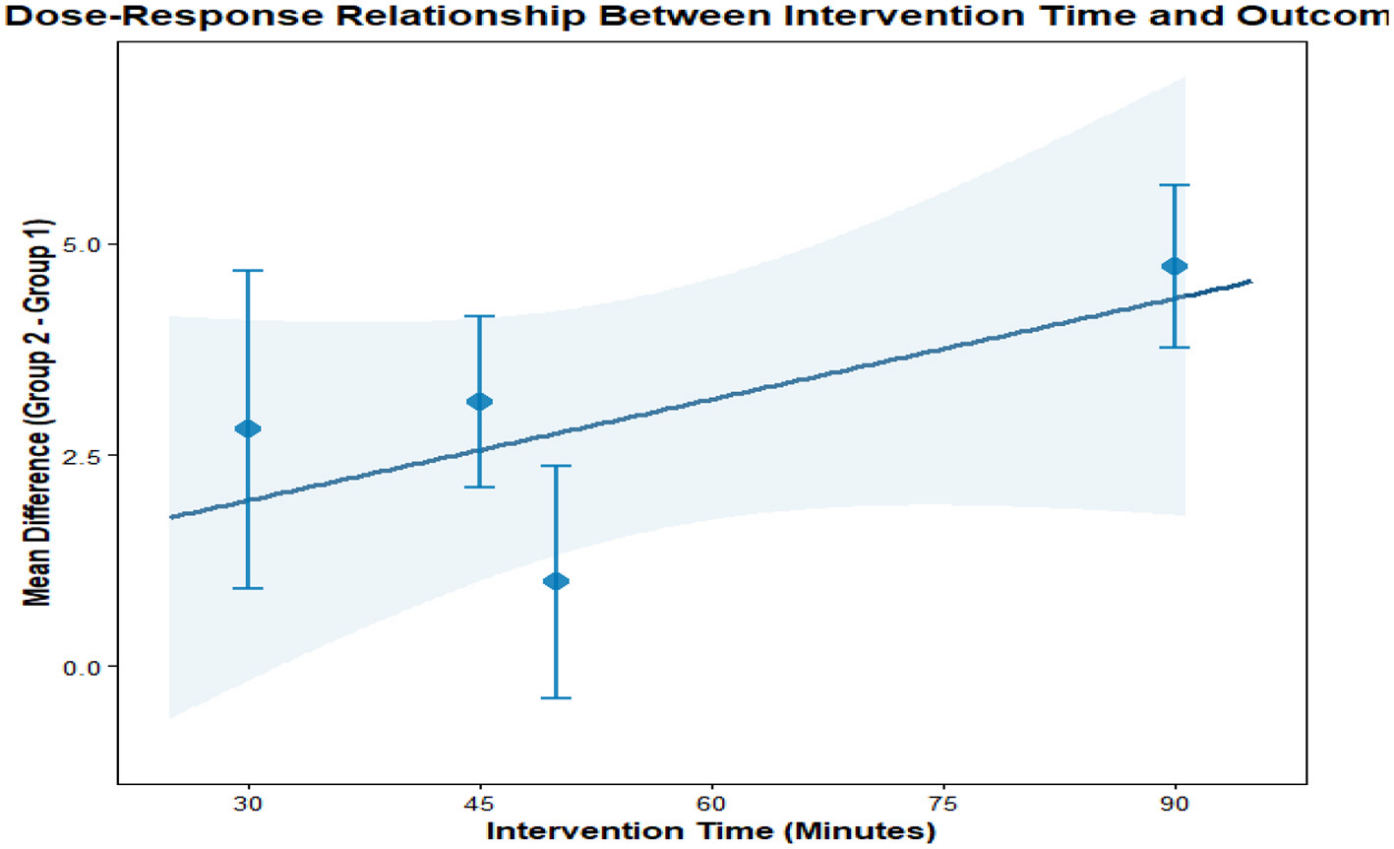
The effect of different intervention durations on mania in patients with bipolar disorder.

This analysis performs a dose-response meta-analysis on depression, anxiety, and mania outcomes in bipolar disorder patients undergoing exercise interventions, focusing on variations in intervention duration, frequency, and single-session length. It classifies intervention frequency as sessions/week, single-session length as minutes/session, and total intervention duration as weeks. Results demonstrate a linear relationship between intervention frequency and both anxiety and mania outcomes (*P* < 0.01): as weekly intervention frequency increases, scores for anxiety and mania symptoms rise significantly—meaning higher intervention frequency correlates with more severe symptoms. Specifically, each additional weekly session elevates the effect size for aggravated anxiety symptoms by 1.5760 (95% CI: 0.7886–2.3635, *P* < 0.0001) and boosts the effect size for exacerbated mania symptoms by 0.7484 (95% CI: 0.4154–1.0815, *P* < 0.0001). Further examination of the dose-response curves shows that within the intervention frequency range included in the analysis (2–7 sessions/week), symptom scores increase steadily as frequency rises. No significant associations exist between depression outcomes and intervention duration, frequency, or single-session length (all *P* > 0.05); similarly, the associations between anxiety/mania outcomes and intervention duration or single-session length hold no statistical significance (all *P* > 0.05). Detailed results are provided in Table 6 and Figures 10–18.

**Table 6:** Meta analysis of dose-response of different exercise intervention for bipolar disorder.

**Index**	**Variety**	**Estimate**	**SE**	***Z***	***P***	**95% CI**
Depression	Weeks	−0.0069	0.1525	−0.0451	0.9641	(−0.3058, 0.2921)
	Frequency	0.3674	1.2658	0.2902	0.7717	(−2.1136, 2.8483)
	Time	0.0133	0.0631	0.2106	0.8332	(−0.1103, 0.1369)
Anxiety	Weeks	0.0547	0.1496	0.3658	0.7145	(−0.2385, 0.3480)
	Frequency	1.5760	0.4018	3.9227	< 0.0001	(0.7886, 2.3635)
	Time	0.0832	0.1014	0.8204	0.4120	(−0.1155, 0.2818)
Mania	Weeks	0.0451	0.0617	0.7322	0.4640	(−0.0757, 0.1660)
	Frequency	0.7484	0.1699	4.4043	< 0.0001	(0.4154, 1.0815)
	Time	0.0399	0.0319	1.2479	0.2121	(−0.0227, 0.1025)

### FDR correction

**Figure 19:**
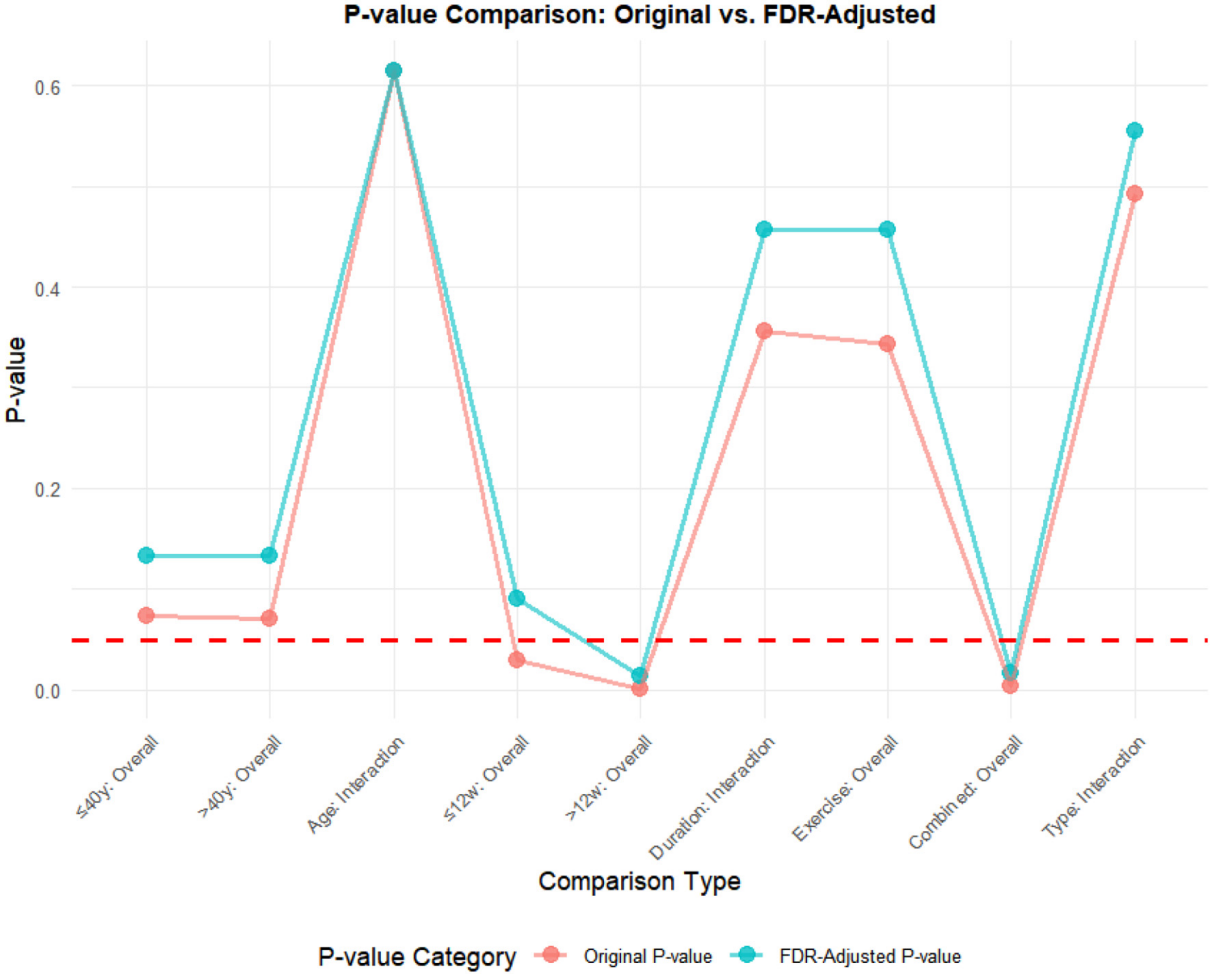
FDR correction chart for depression outcome indicator.

**Figure 20:**
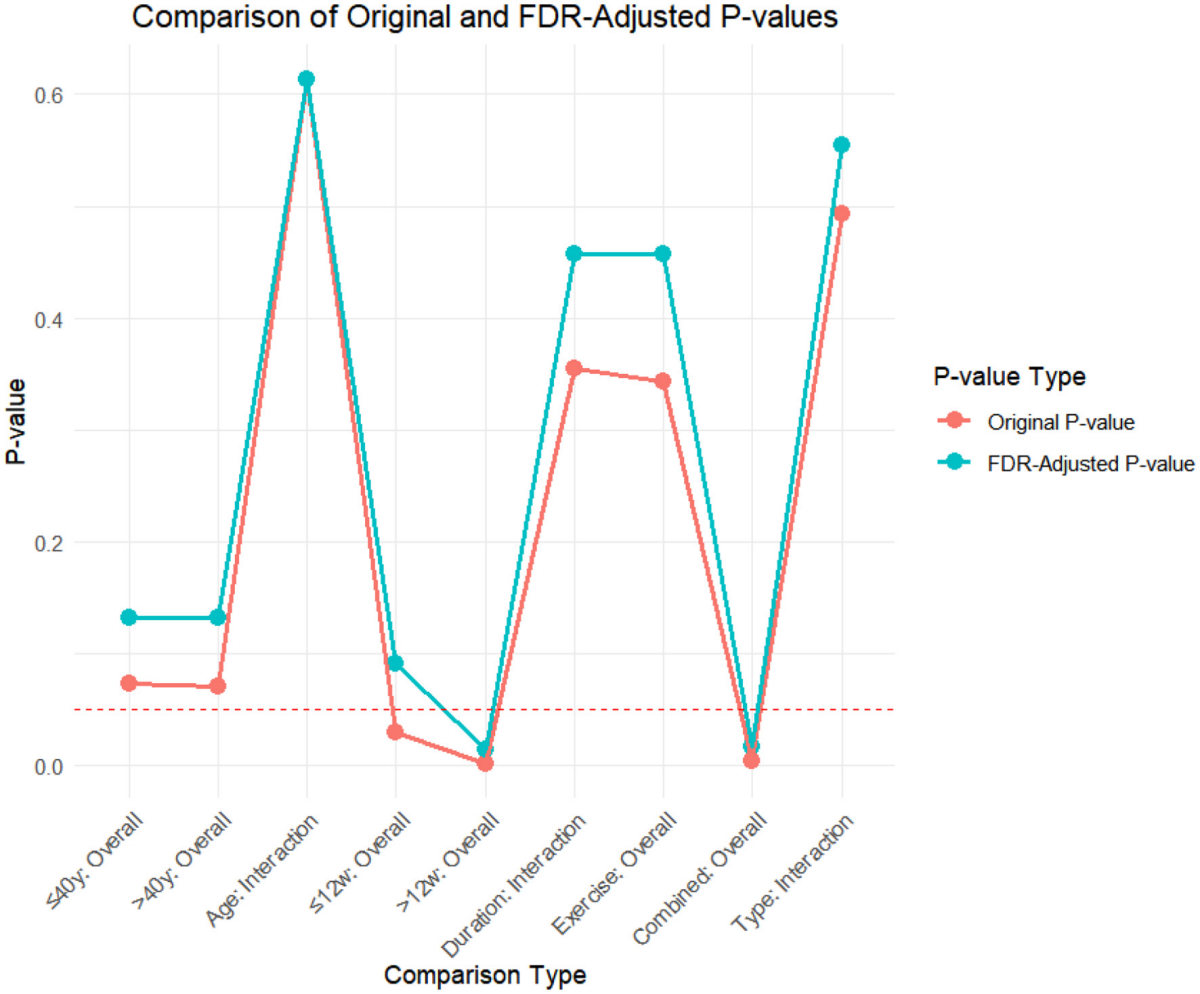
Anxiety outcome measure FDR correction chart.

**Figure 21:**
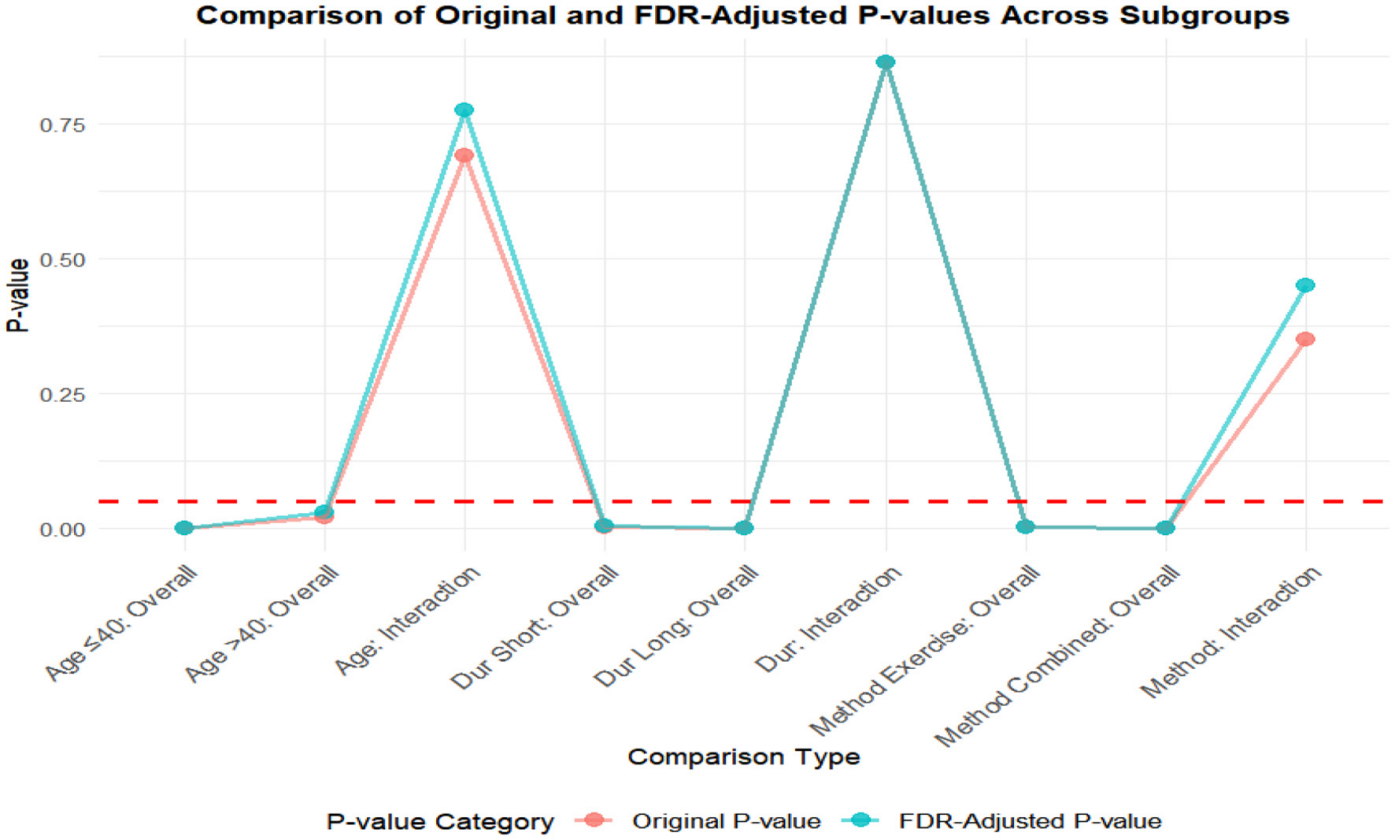
Frenzy outcome measure FDR correction chart.

**Figure 22:**
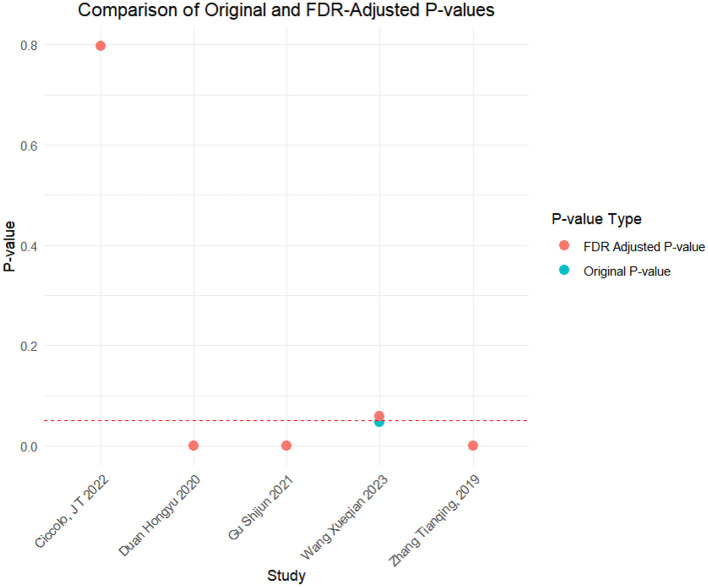
FDR correction chart for health questionnaire outcome indicators.

**Figure 23:**
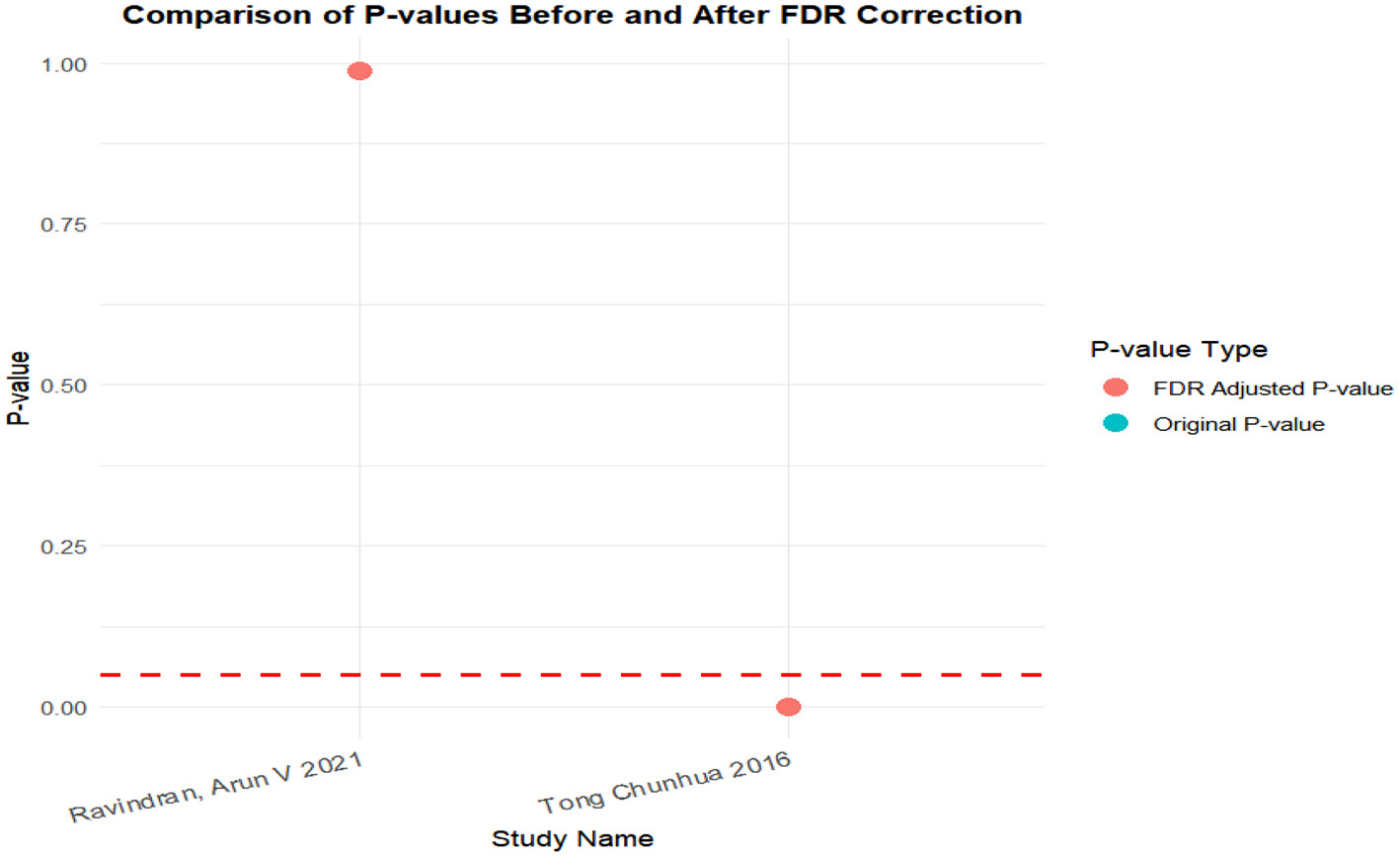
FDR correction chart of life questionnaire outcome indicators.

**Figure 24:**
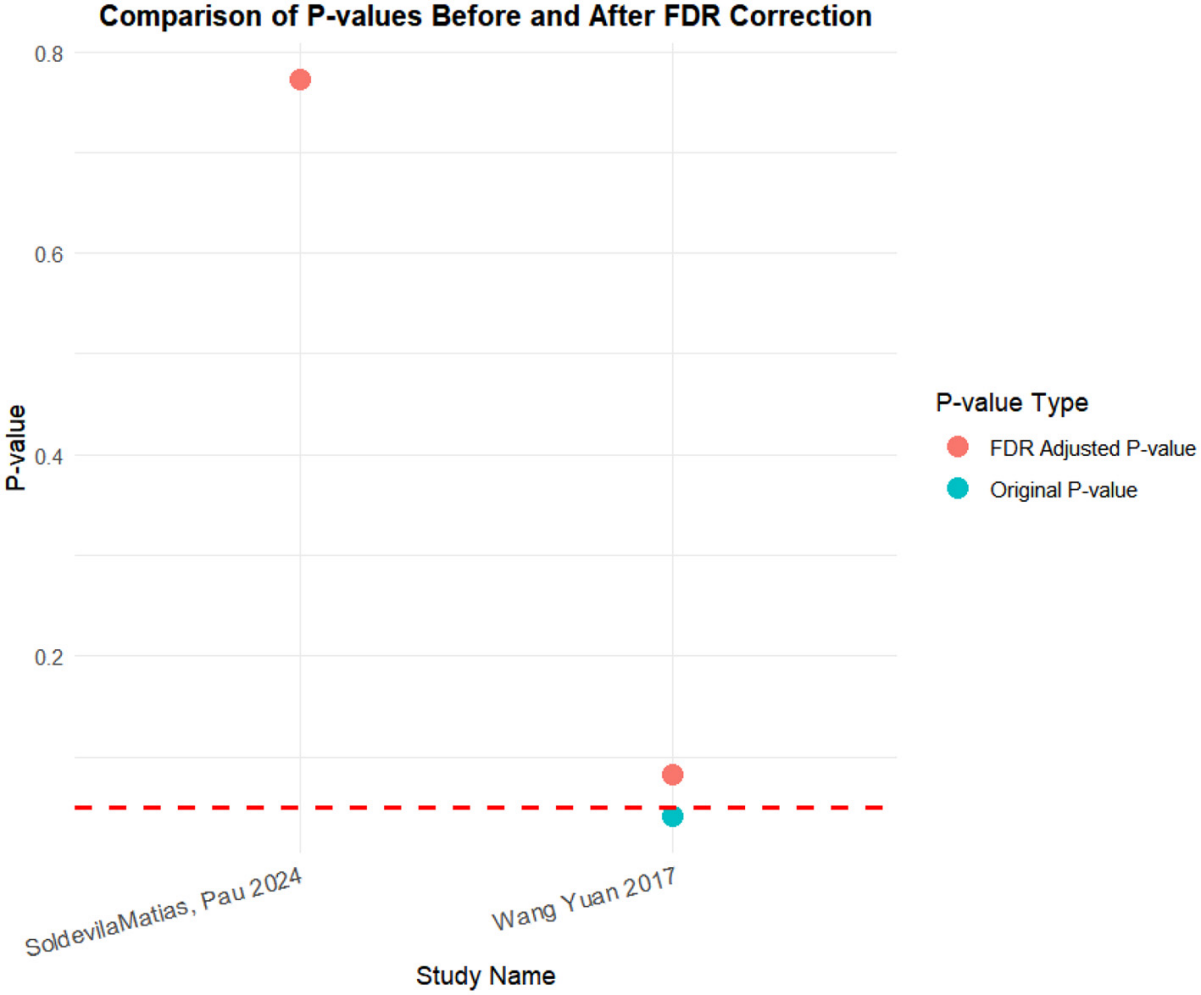
FDR correction chart for systolic blood pressure outcome indicator.

**Figure 25:**
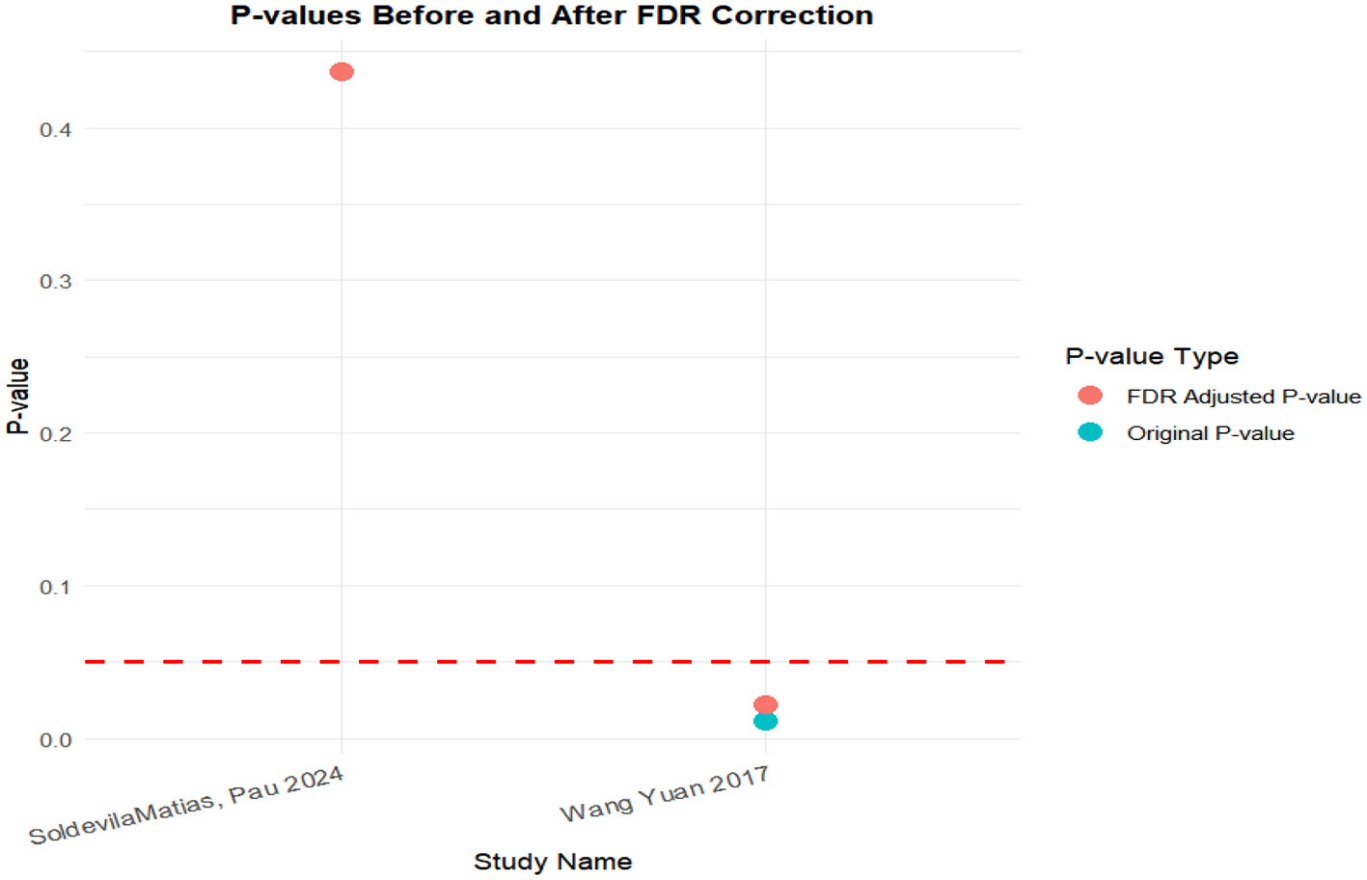
FDR correction chart for diastolic blood pressure outcome measure.

A meta-analysis was performed via R software to assess the effects of psychological and exercise interventions on psychiatric symptoms (depression, anxiety, mania) as well as physiological and quality-of-life outcomes (health questionnaire, life questionnaire, systolic blood pressure, and diastolic blood pressure). To control for type *I* errors resulting from multiple comparisons, the Benjamini–Hochberg procedure was applied for false discovery rate (FDR) correction, with adjusted *P* < 0.05 considered statistically significant. The analysis revealed that, for depressive symptoms, only combined interventions lasting more than 12 weeks had statistically significant effects (adjusted *P* = 0.0146). In contrast, short-term and exercise-only interventions had no significant effects, and age did not moderate the outcomes. For anxiety and mania, the intervention effects were consistent across subgroups: both short-term and long-term and exercise-only and combined interventions yielded significant improvements, with no significant subgroup interaction effects observed. With respect to health questionnaire outcomes, three studies reported significant effects (Gu et al., 2021; Duan and Li, 2020; Zhang et al., 2019), whereas two studies did not. For the life questionnaire, only the study by Tong showed a significant effect. Among blood pressure indicators, a significant reduction was found only in diastolic blood pressure (adjusted *P* = 0.0224), whereas systolic blood pressure remained unaffected. Overall, the intervention efficacy varied according to outcome measures and subgroup characteristics, emphasizing the importance of individualized clinical strategies. The analytical framework adhered to methodological standards consistent with leading SCI-indexed meta-analysis publications. The detailed results are presented in Table 7 and Figures 19–25.

**Table 7:** FDR correction table for the effects of different exercise interventions on bipolar disorder.

**Index**	**Comparison type**	**Original *P*-value**	**Corrected *P*-value**	**Conclusion (after correction)**
Depression	≤ 12 weeks	0.0303	0.0909	Non-significant
	>12 weeks	0.0016	0.0146	Significant
	Duration subgroup interaction effect	0.355	0.457	Non-significant
	Overall effect of simple exercise group	0.344	0.457	Non-significant
	The overall effect of the joint intervention group	0.0037	0.0168	Significant
	Mode subgroup interaction effect	0.493	0.554	Non-significant
	≤ 40 years old	0.0737	0.133	Non-significant
	>40 years old	0.0712	0.133	Non-significant
	Age	0.614	0.614	Non-significant
Anxiety	≤ 40 years old	0	0.0001	Significant
	>40 years old	0.0202	0.0303	Significant
	Age	0.690	0.776	Non-significant
	≤ 12 weeks	0.0021	0.0038	Significant
	>12 weeks	0	0	Significant
	Duration subgroup interaction effect	0.865	0.865	Non-significant
	Overall effect of simple exercise group	0.0009	0.002	Significant
	The overall effect of the joint intervention group	0	0.0001	Significant
	Mode subgroup interaction effect	0.350	0.450	Non-significant
Mania	≤ 40 years old	0	0.0001	Significant
	>40 years old	0.0202	0.0303	Significant
	Age	0.690	0.776	Non-significant
	≤ 12 weeks	0.0021	0.0038	Significant
	>12 weeks	0	0	Significant
	Duration subgroup interaction effect	0.865	0.865	Non-significant
	Overall effect of simple exercise group	0.0009	0.002	Significant
	The overall effect of the joint intervention group	0	0.0001	Significant
	Mode subgroup interaction effect	0.350	0.450	Non-significant
Health questionnaire	Wang Xueqian 2023	0.0467	0.0584	Non-significant
	Gu Shijun 2021	0	0	Significant
	Duan Hongyu 2020	0	0	Significant
	Zhang Tianqing, 2019	0	0	Significant
	Ciccolo, J T 2022	0.797	0.797	Non-significant
Life questionnaire	Tong Chunhua 2016	0.000001	0.000002	Significant
	Ravindran, Arun V 2021	0.988	0.988	Non-significant
Systolic pressure	Wang Yuan 2017	0.0410	0.0821	Non-significant
	SoldevilaMa, Pau 2024	0.772	0.772	Non-significant
Diastolic pressure	Wang Yuan 2017	0.0112	0.0224	Significant
	SoldevilaMa 2024	0.437	0.437	Non-significant

### Sensitivity analysis

A sensitivity analysis was conducted on the baseline characteristics of the control groups for depression, anxiety, and mania outcome measures via the leave-one-out method. As presented in Table 8, both the overall and subgroup analyses for depression and anxiety—after excluding either exercise intervention or usual care controls—showed *I*^2^ values below 50% and *P* values above 0.05, indicating low heterogeneity and stable results. For mania outcomes, heterogeneity decreased (*I*^2^ < 50%, *P* > 0.05) when usual care controls were excluded, whereas excluding exercise intervention controls increased heterogeneity (*I*^2^ > 50%, *P* < 0.05). These findings suggest that the exclusion of exercise intervention controls affected the pooled results for mania, whereas the depression and anxiety outcomes remained stable regardless of which control type was excluded.

**Table 8:** Sensitivity analysis of baseline characteristics of different exercise interventions on bipolar disorder.

**Index**	**Compare types**	**SMD**	**95% CI**	***P***	***I*^2^ %**	***P***
Depression	Exclude exercise intervention	0.020	(−0.111, 0.151)	0.766	0.0	0.622
	Exclude routine care	−0.091	(−0.319, 0.138)	0.437	0.0	0.0.412
	Total	−0.007	(−0.121, 0.106)	0.897	0.0	0.702
Anxiety	Exclude exercise intervention	0.053	(−0.111, 0.217)	0.526	0.0	0.557
	Exclude routine care	−0.095	(−0.323, 0.134)	0.417	0.0	0.455
	Total	0.003	(−0.130, 0.136)	0.968	0.0	0.589
Mania	Exclude exercise intervention	−0.168	(−0.354, 0.017)	0.075	66	0.012
	Exclude routine care	−0.100	(−0.328, 0.128)	0.390	0.0	0.328
	Total	−0.141	(−0.285, 0.003)	0.055	55.9	0.026

**Figure 26:**
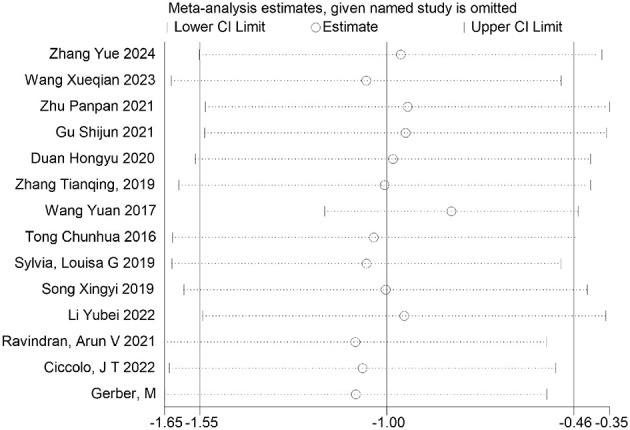
Sensitivity analysis of depression outcomes indicators.

**Figure 27:**
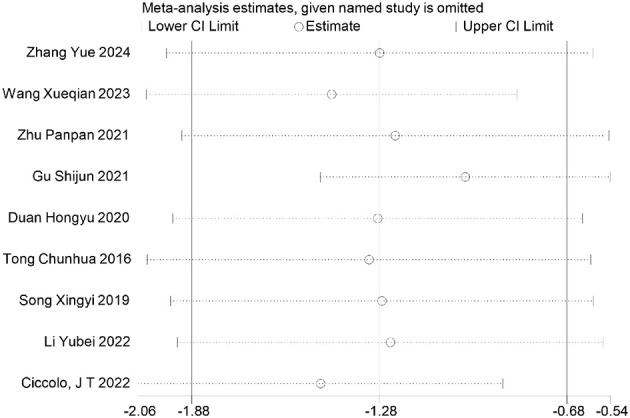
Sensitivity analysis of anxiety outcomes indicators.

**Figure 28:**
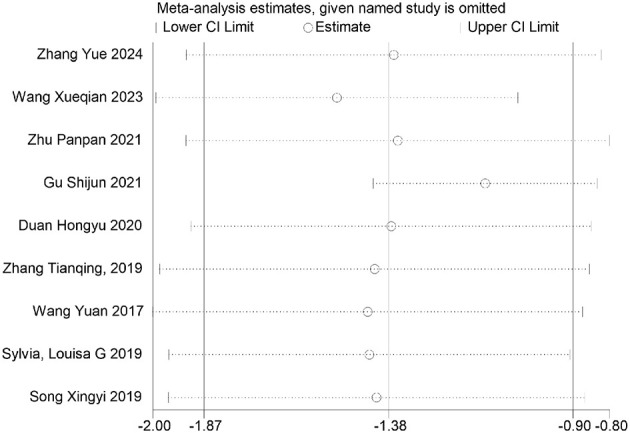
Sensitivity analysis of manic outcomes indicators.

**Figure 29:**
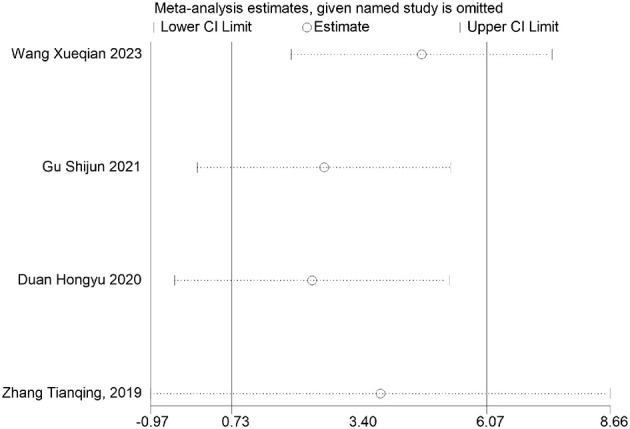
Sensitivity analysis of health questionnaire outcomes indicators.

**Figure 30:**
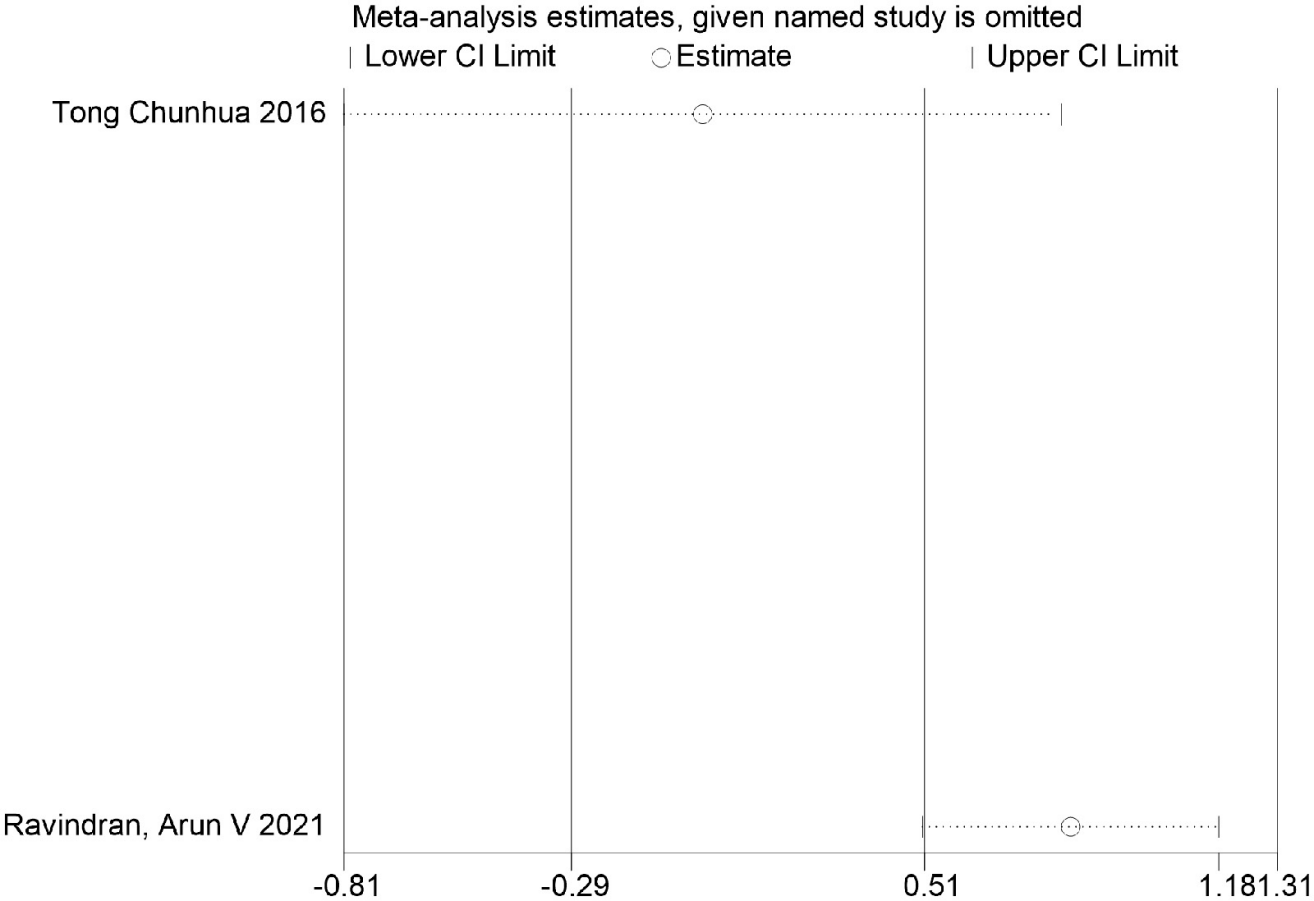
Sensitivity analysis of life questionnaire results indicators.

**Figure 31:**
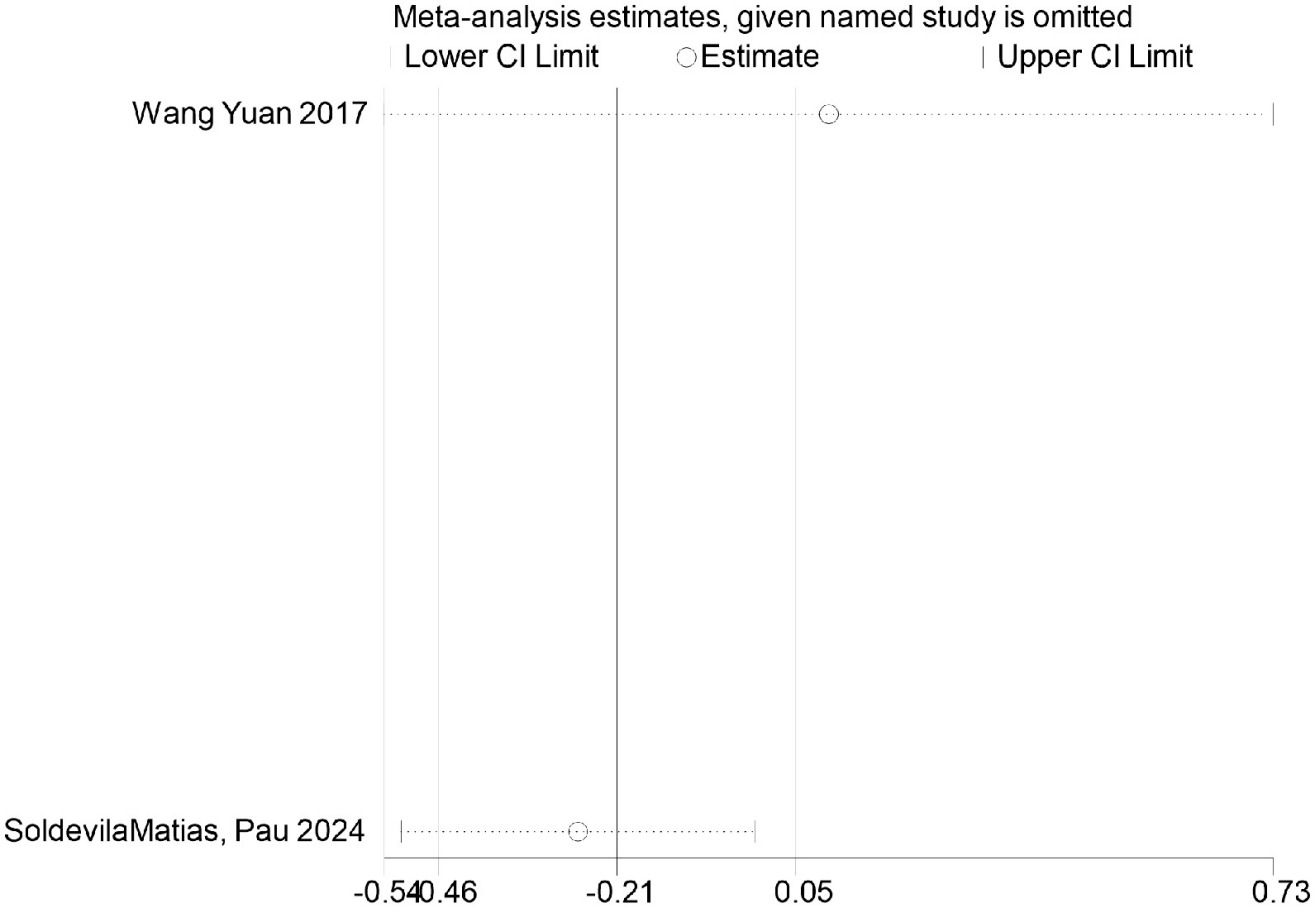
Sensitivity analysis of outcome indicators for systolic blood pressure.

**Figure 32:**
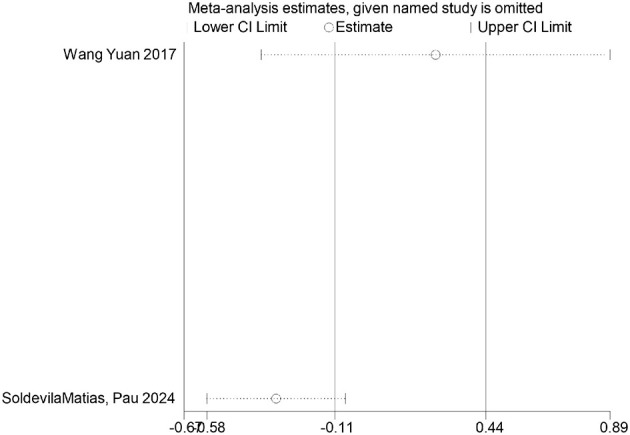
Sensitivity analysis of diastolic blood pressure outcome indicators.

Furthermore, sensitivity analyses performed via Stata software (Figures 26–32) confirmed the robustness and stability of the meta-analysis results for depression, anxiety, and mania outcomes. In contrast, heterogeneity was observed in the analyses of health questionnaire, life questionnaire, systolic blood pressure, and diastolic blood pressure outcomes.

### Publication bias test

**Figure 33:**
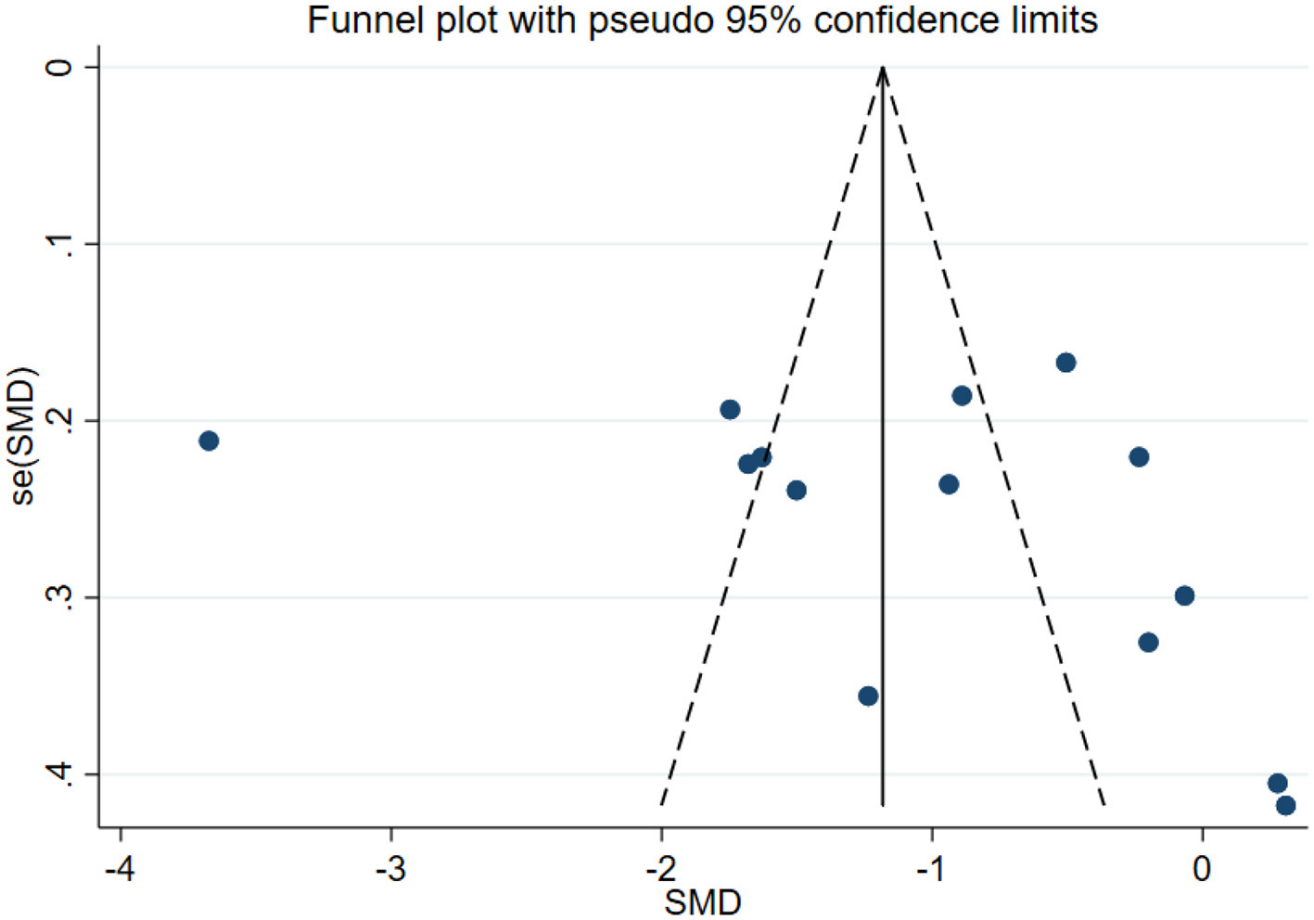
Depression funnel plot.

**Figure 34:**
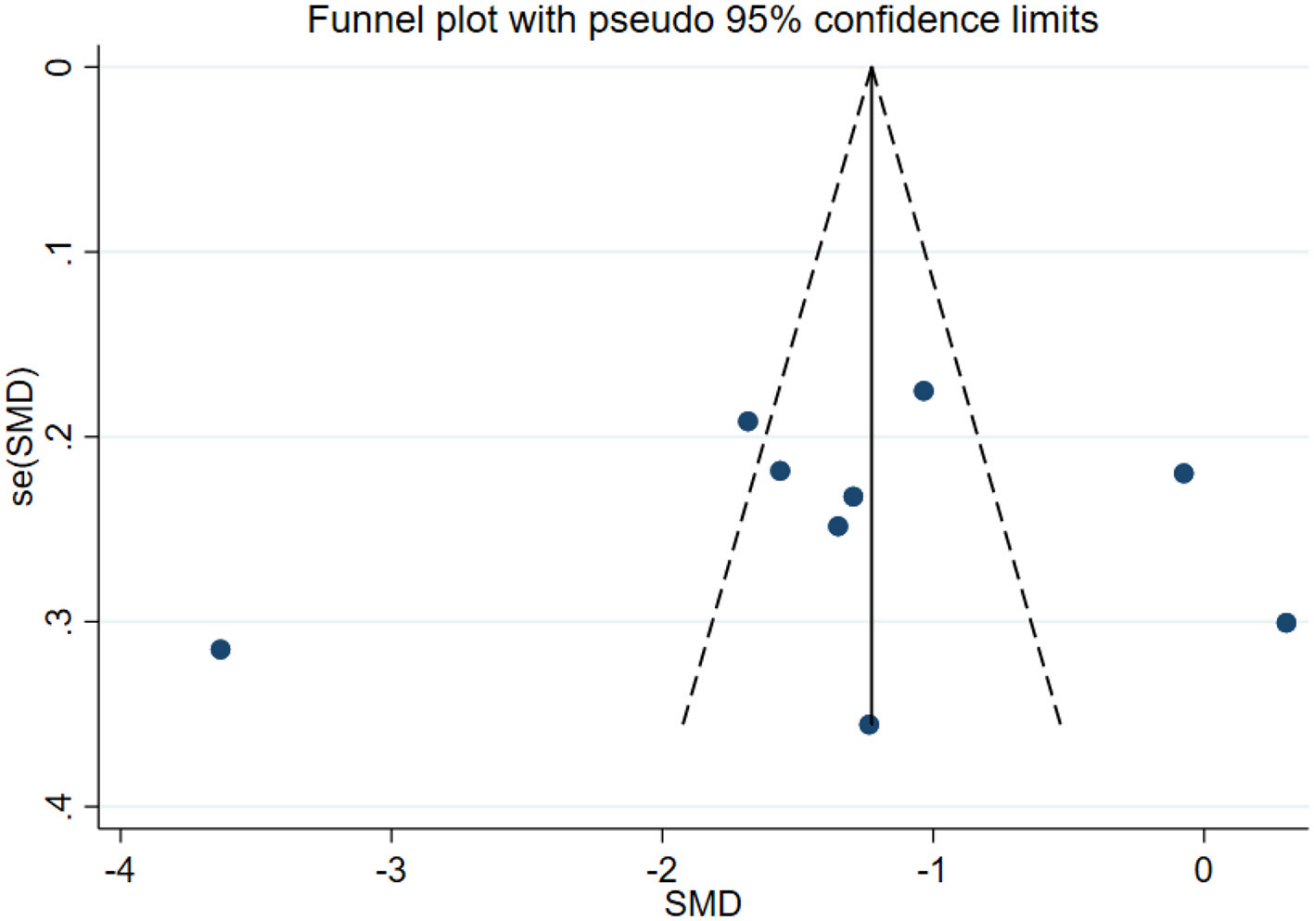
Anxiety funnel plot.

**Figure 35:**
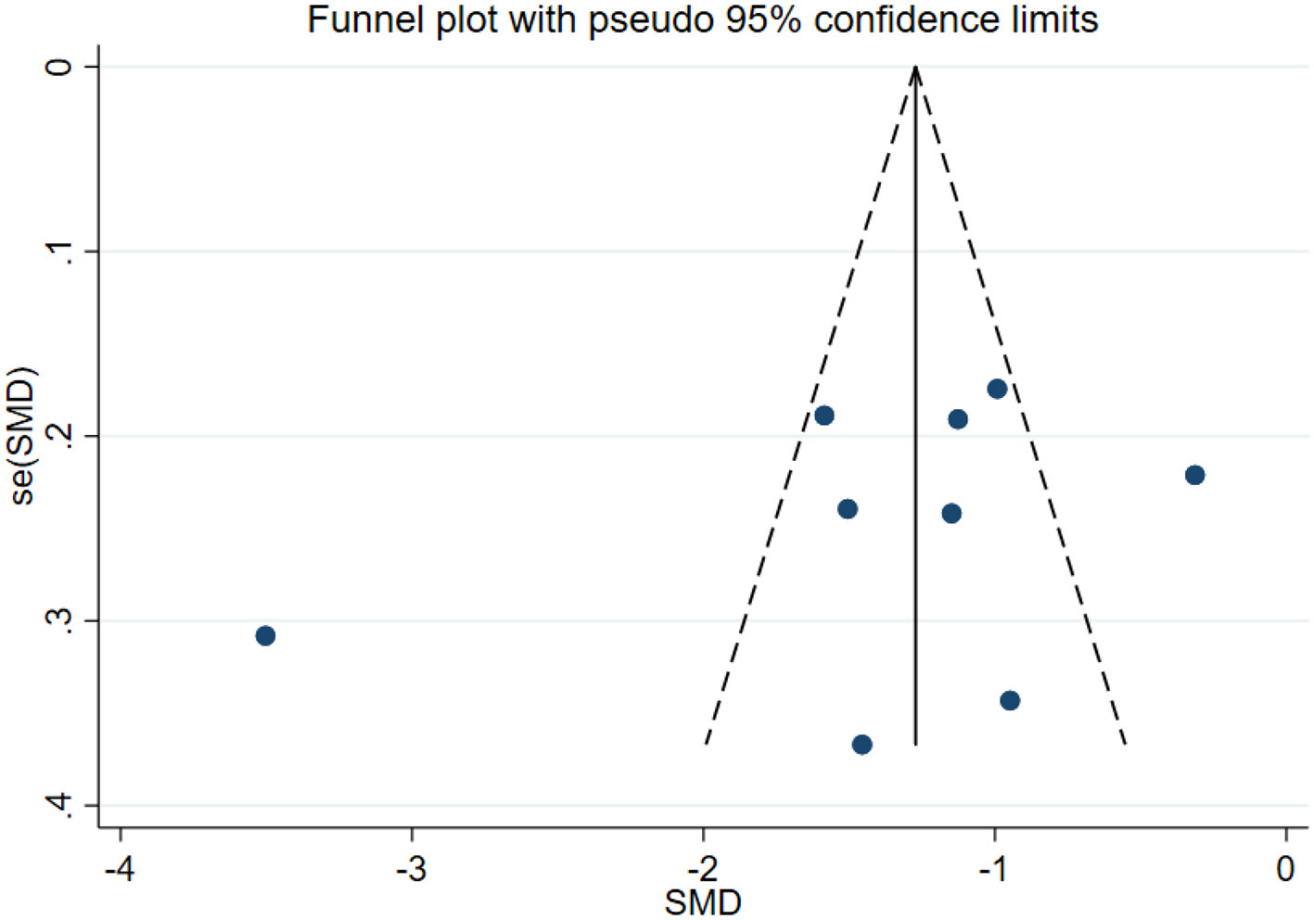
Maniac funnel plot.

**Figure 36:**
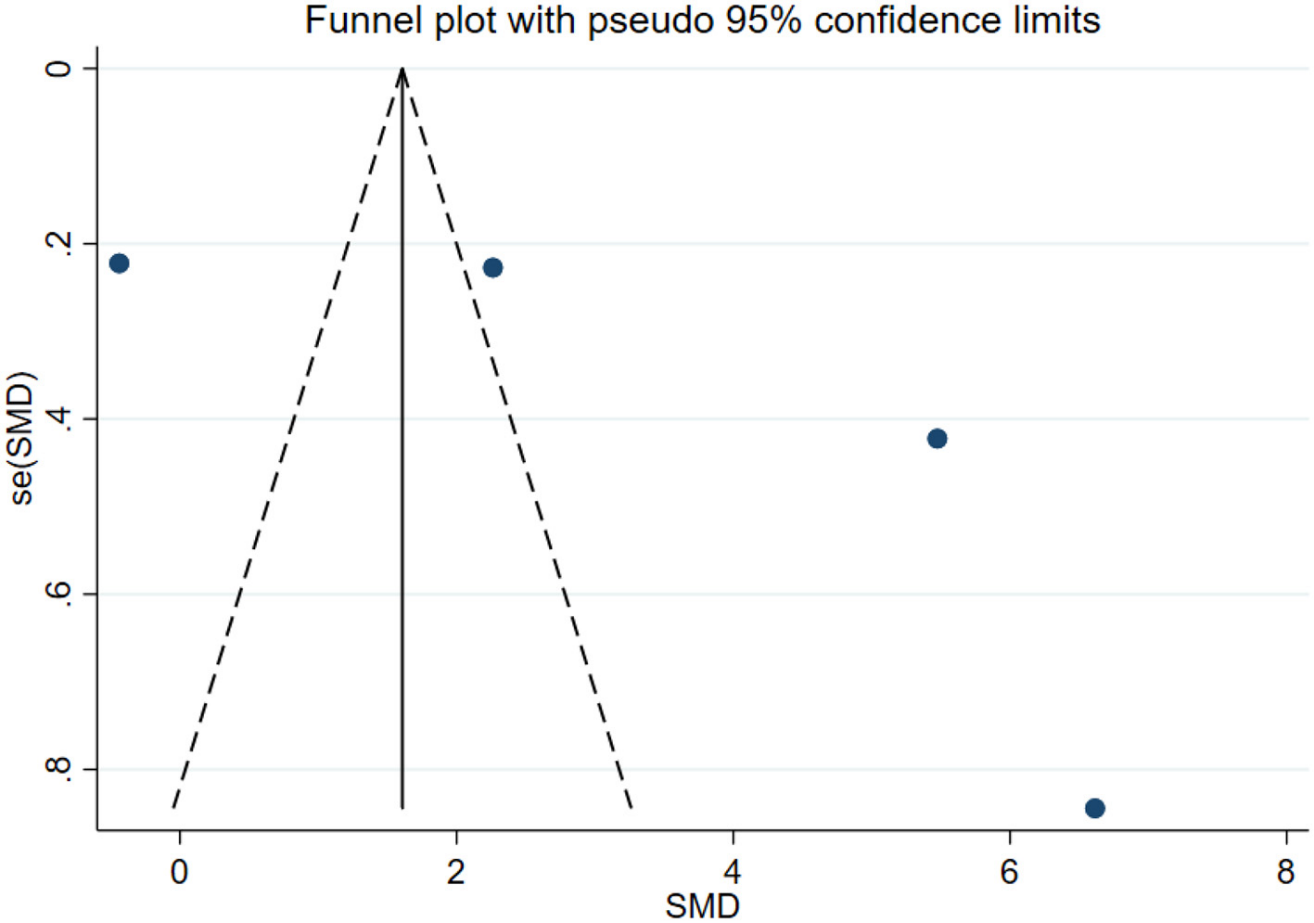
Health questionnaire funnel chart.

**Figure 37:**
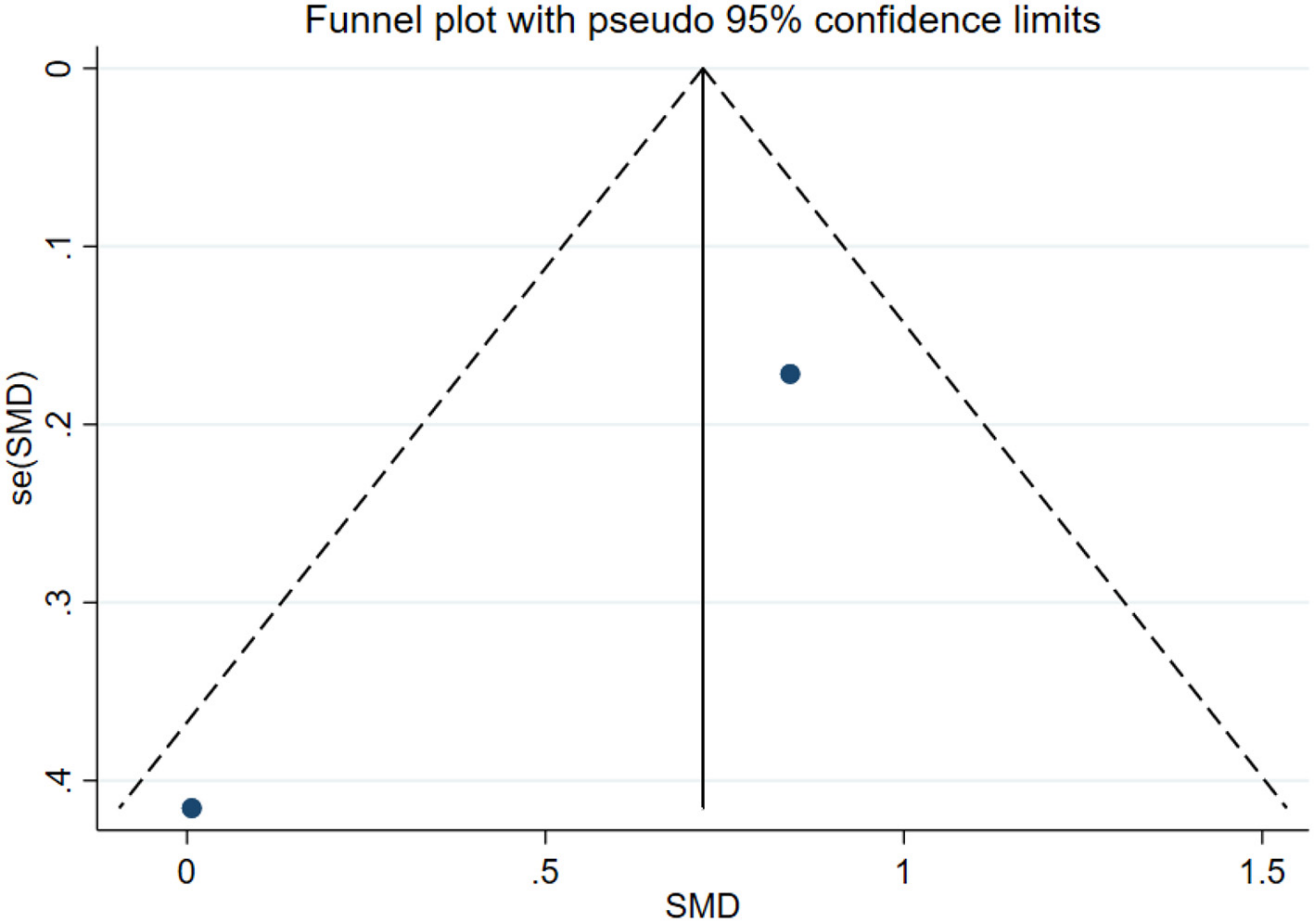
Funnel chart of life questionnaire.

**Figure 38:**
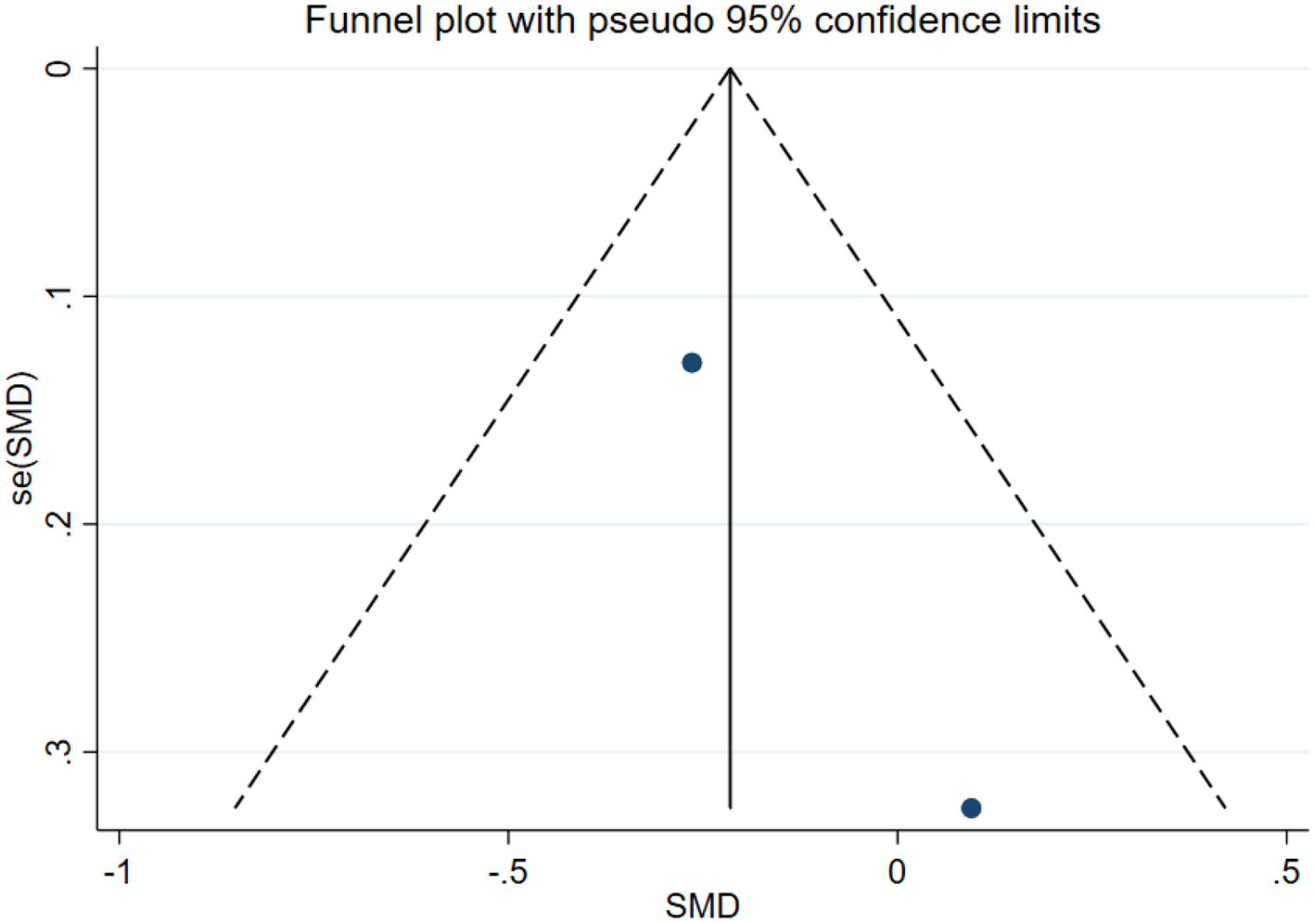
Shrinkage pressure funnel diagram.

**Figure 39:**
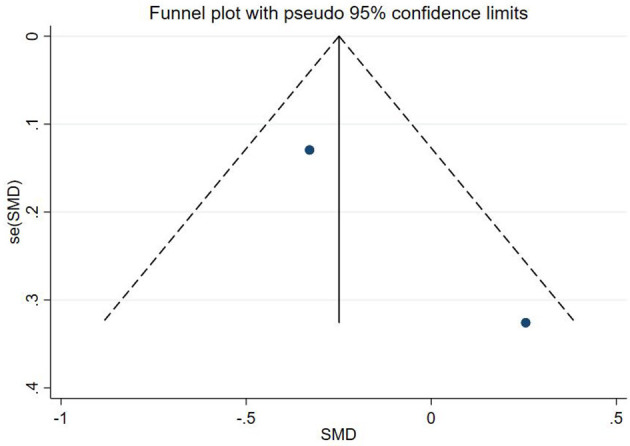
Funnel plot of diastolic blood pressure.

In this study, publication bias for the outcomes of depression, anxiety, mania, health questionnaire, life questionnaire, systolic blood pressure, and diastolic blood pressure with exercise intervention for bipolar affective disorder was assessed via Egger's test, Begg's test, and funnel plots in Stata software. The results indicated that there was no publication bias for any of the tested outcomes (*P* > 0.05), as shown in Table 9, Appendix 5 and Figures 33–39.

**Table 9:** Publication bias test of different exercise interventions on bipolar disorder.

**Outcome indicators**	**Egger's test (*P* value)**	**Begg's test (*P* value)**
Depression	0.296	0.443
Anxiety	0.729	1.000
Mania	0.357	0.602
Health questionnaire		1.000
Life questionnaire		1.000
Systolic blood pressure		1.000
Diastolic blood pressure		1.000

### Meta-regression analysis

Meta-regression analysis was initially conducted to evaluate the baseline characteristics of the outcome measures in the control groups of the included studies, with disease duration included as a covariate in the regression model. The results, presented in Table 10, indicated that baseline characteristics, including age, disease duration, and outcome measure values, did not significantly affect the intervention effect size (*P* > 0.05 for all), suggesting that these control-related factors did not introduce any confounding bias.

**Table 10:** Baseline feature regression analysis of the effects of different exercise interventions on patients with bipolar disorder.

**Index**	**_ES**	**(95% CI)**	***t***	***P***
Age	Cons	(−0.2804308, 0.2819079)	0.01	0.996
Course of disease	Cons	(−0.3573461, 0.1479795)	−1.07	0.336
Depression	Course	(−0.4056817, 0.1849434)	−0.82	0.428
	Cons	(−0.2596554, 0.5201271)	0.74	0.478
Anxiety	Course	(−0.4866848, 0.1913241)	−1.03	0.337
	Cons	(−0.2814031, 0.6830492)	0.98	0.358
Mania	Course	(−0.7065478, 0.9643378)	0.38	0.719
	Cons	(−1.51073, 0.7946406)	−0.76	0.476
Health questionnaire	Cons	(−1.589359, 1.85158)	0.97	0.510
Life questionnaire	Cons	(−2.050785, 1.836685)	−0.70	0.611
Systolic blood pressure	Cons	(−1.528338, 1.613264)	0.34	0.789
Diastolic blood pressure	Cons	(−1.621928, 1.519425)	−0.41	0.750

Another meta-regression analysis was subsequently performed to explore the sources of heterogeneity across the studies. The combined effect size was set as the dependent variable, with covariates such as intervention type (exercise only, exercise combined with psychological therapy, and exercise combined with mindfulness training), intervention duration (12, < 12, >12 weeks), intervention frequency (2 times/week, >2 times/week), and session duration ( ≤ 60, >60 min). This model simultaneously analyzed outcomes for depression, anxiety, and mania in patients with bipolar disorder, as well as outcomes for the health questionnaire, life questionnaire, systolic blood pressure, and diastolic blood pressure. The results revealed that, except for the intervention frequency for the mania outcome (which displayed heterogeneity, *P* < 0.05), no significant sources of heterogeneity were found for the other outcomes (*P* > 0.05). The detailed results are presented in Table 11.

**Table 11:** Regression analysis of the effects of different exercise interventions on patients with bipolar disorder.

**Bipolar disorder index**	**_ES**	**(95% CI)**	***t***	***p***
Depression	W1	(−0.0411753, 0.2607032)	2.31	0.104
	F1	(−2.563228, 0.8739273)	−1.56	0.216
	T1	(−0.063611, 0.0547892)	−0.24	0.828
	T2	(−4.960621, 5.481271)	0.16	0.884
Anxiety	W1	(−2.213095, 2.167396)	−1.31	0.200
	F1	(−28.21811, 26.47476)	−0.41	0.755
	T1	(−1.064191, 1.106514)	0.25	0.845
	T2	(−69.36913, 69.84411)	0.04	0.972
Mania	W1	(−0.036174, 0.1284905)	2.41	0.137
	F1	(−2.522472,−0.0210874)	−4.38	0.048
	T1	(−0.091502, 0.0352337)	−1.91	0.196
	T2	(−3.468648, 5.362455)	0.92	0.454
Health questionnaire		(−1.654998, 8.499089)	2.15	0.121
Life questionnaire		(−4.681288, 5.700804)	1.25	0.430
Systolic blood pressure		(−1.861478, 1.444986)	−1.60	0.356
Diastolic blood pressure		(−3.692943, 3.466301)	−0.40	0.757

### Assessment of evidence quality

The GRADE approach was employed to assess the quality of evidence for the outcome measures included in this study. The analysis revealed that the quality of evidence for depression, anxiety, and mania outcomes was moderate; for the health questionnaire, it was low; and for the systolic blood pressure, life questionnaire, and diastolic blood pressure, it was very low. The detailed results are presented in Appendix 3.

## Discussion

### Mechanisms of intervention for different exercise models

This study employs a meta-analysis approach to systematically investigate the effects of different exercise intervention models on emotional disorders in patients with bipolar affective disorder. The results showed that exercise combined with psychotherapy or exercise combined with mindfulness training significantly improved depression and anxiety symptoms, whereas exercise alone was effective only in alleviating manic symptoms. This phenomenon is closely related to the multidimensional mechanisms of action: patients experiencing depressive episodes are often closely associated with dysregulation of the hypothalamic–pituitary–adrenal (HPA) axis and are frequently accompanied by symptoms such as low mood, reduced motivation, and negative thinking and behavior (Gerber et al., 2020). Compared with those receiving conventional psychological interventions without yoga practices, the group receiving 8 weeks of yoga intervention (with a breathing rate of 40–60 breaths per minute) presented significantly alleviated depressive symptoms as measured by the Montgomery-Åsberg Depression Rating Scale (MADRS; Hofmann et al., 2010). Moreover, compared with drug intervention without exercise, 8 weeks of moderate-intensity aerobic exercise training (twice a week, 50 min per session) was more effective at improving emotional disorder symptoms in patients, as indicated by the Hamilton Depression Rating Scale, Hamilton Anxiety Rating Scale, and Youngmania Rating Scale scores (*P* < 0.01; Wang, 2023), highlighting the beneficial effects of exercise intervention in bipolar disorder patients. From a mechanistic perspective, yoga and aerobic exercise activate the afferent fibers of the vagus nerve, affecting the autonomic nervous system, neuroendocrine system, and limbic system circuits and regulating cortisol levels in the adrenal axis, thereby alleviating depressive symptoms (Ciccolo et al., 2022). Mindfulness training and psychotherapy can also activate the default mode network and the prefrontal–limbic system connection to regulate the HPA axis, helping patients improve concentration, slow cognitive processing speed, normalize cortisol secretion, reduce dependence on medication and enhance emotional regulation capabilities to improve emotional disorders (Cotton et al., 2016). Psychological interventions assist patients in building self-confidence through mental support, counseling, guidance, encouragement, and reassurance. Physical activity improves communication skills and facilitates the restoration of social functioning. The combination of both interventions enables patients to express and manage their emotions, yielding long-term benefits. This approach enhances medication adherence, reduces the risk of relapse, and improves the ability to live independently, thereby enhancing overall quality of life. Further research comparing exercise combined with psychotherapy or mindfulness training interventions to exercise-only, psychotherapy-only, or no-exercise control groups revealed significant improvements in the SAS, SDS, BMRS scores and corresponding quality of life and mental health scores after 2–9 months of intervention (*P* < 0.05; Zhang et al., 2024). Exercise interventions significantly reduce negative emotions, such as depression and anxiety, in patients with bipolar disorder. Furthermore, incorporating psychological interventions, such as mindfulness and meditation, into exercise programs further alleviates these negative emotions and enhances positive feelings. However, it is important to note that current research on the combination of exercise with psychological or mindfulness interventions—specifically those involving 2–3 sessions per week, each lasting ≤ 90 min and lasting 12 weeks or more—has insufficient sample sizes, resulting in reduced statistical power. The clinical effectiveness of this combined approach requires further validation through larger, high-quality studies.

From a neuroendocrine perspective, 15 min of aerobic exercise regulates cortisol levels, and when combined with psychological interventions, this further influences the neurotransmitter system. Previous studies have shown that moderate-intensity exercise reduces the concentration of high vanillic acid (HVA), a dopamine metabolite, in cerebrospinal fluid while increasing phenylacetic acid secretion in urine, releasing plasma arachidonoyl amides, and optimizing neurotransmission in brain regions related to the pathophysiology of the disease (Sparling et al., 2003). Mindfulness training and psychotherapy reshape patients' cognitive frameworks toward self and emotions (Hofmann et al., 2010), optimizing the effects of neurotransmitters and forming a synergistic mechanism of exercise-neuroregulation-cognitive restructuring.

### Neurobiological basis of exercise intervention timing characteristics

At the intervention cycle level, both ≤ 12 and >12 weeks of exercise interventions significantly improved depression, anxiety, and mania symptoms, which are closely related to the dynamic regulation of inflammatory factors. High-intensity exercise for 9–12 weeks can significantly reduce C-reactive protein (CRP) levels, whereas 3–12 months of aerobic exercise and resistance training can regulate proinflammatory factors such as interleukin-6 (IL-6) and tumor necrosis factor-alpha (TNF-α), creating an anti-inflammatory environment (Rose et al., 2021). Differences in the frequency and duration of weekly interventions are also closely related to neurobiology. Exercise twice a week significantly improves anxiety symptoms, whereas exercise interventions more than twice a week are more beneficial for improving manic and depressive symptoms. This is associated with the induction of neuroplasticity. Frequent exercise was associated with mania (*P* = 0.012; Wang, 2023). Prolonged high-intensity exercise can result in chronic increases in catecholamine and cortisol levels, as well as heightened fatigue, which may contribute to increased chronic inflammation and potentially trigger or worsen manic symptoms. For example, an 8-week moderate-intensity intermittent exercise program (5 sessions per week, 45 min per session) resulted in significant differences in manic symptoms compared with the usual care group with no exercise intervention, both within and between groups (*P* > 0.05; Wang, 2023). In contrast, prolonged endurance exercise may reduce manic symptoms by inhibiting the activity of T-helper 1 (Th1) cells, enhancing Th2 function, stimulating the secretion of brain-derived neurotrophic factor (BDNF) and insulin-like growth factor 1 (IGF-1), and improving hippocampal function (Cotman and Berchtold, 2007; Liu et al., 2017). Experimental studies have demonstrated that a 12-week walking program (3 sessions per week, 60 min per session) significantly affects markers such as IL-6, low-density lipoprotein (LDL), and SBP.

Single-session interventions lasting more than 60 min significantly improved manic and anxiety symptoms. Long-duration endurance activities increase cardiovascular load, stimulate the release of protective factors, activate afferent fibers of the vagus nerve, and regulate autonomic nervous and limbic system functions (Thomson et al., 2015). Low-intensity endurance exercise for 60–90 min effectively reduces the accumulation of reactive carbonyl derivatives (RCDs) caused by aging, enhances protease complex activity, promotes neuroplasticity, improves emotional disorders, and increases wellbeing (Radak et al., 2001). Research has shown that compared with the conventional treatment group, the low-intensity exercise group, which had a 3-month intervention period and exercised five times a week with sessions lasting 30–60 min, had significantly improved total cholesterol, triglyceride, and depression levels (*P* < 0.05).

### Integrated strategies for exercise intervention

Given the generally low levels of physical activity in patients with bipolar affective disorder, a survey of 60 bipolar patients revealed that approximately 78% of patients were sedentary and did not meet the standard of 150 min of moderate-intensity or vigorous exercise per week (Thomson et al., 2015). Additionally, many patients have comorbid metabolic syndrome and low self-efficacy, suggesting that exercise interventions must be integrated with multidimensional strategies. Rhythmic aerobic exercise (such as swimming and running) can exert a sedative effect by activating the γ-aminobutyric acid (GABA) system and regulating mood (Alsuwaidan et al., 2009). A 12-week exercise program with moderate-intensity aerobic exercise three times per week for 60 min is recommended. This plan not only significantly improves weight and health behaviors but also enhances cognitive function by regulating dopaminergic neurotransmission. Alternatively, low-intensity group exercises (such as group walking or calisthenics) 2–3 times per week for 30 min may reduce depression by promoting social interaction and synergistic exercise effects (Wang et al., 2017).

The integration of psychological interventions is key to reducing patients' medication dependence in bipolar affective disorder patients. Cognitive behavioral therapy and mindfulness training can reshape patients' perceptions and coping strategies toward emotions, reducing overactivation of the HPA axis. When combined with exercise interventions, they can regulate neurotransmitters and inflammatory factors. In clinical practice, the “exercise prescription + psychological skills training” model can help patients develop regular exercise habits, enhance self-efficacy, and improve the social support system through social interaction, ultimately achieving improved cardiovascular metabolic risk and emotional functioning.

## Conclusion

The findings of the study indicate that, on the basis of low- to moderate-quality evidence, combined exercise and psychological or mindfulness interventions conducted for 12 weeks or longer, with a frequency of two to three sessions per week and a duration of no more than 90 min per session, exert positive effects on negative emotions—including depression, anxiety, and mania—as well as on related measures such as health and life questionnaire scores in adolescents with bipolar disorder. The effects of exercise interventions on emotional disturbances in this population appear to be closely associated with the type, duration, frequency, and length of the intervention. Nevertheless, variations in outcomes across studies suggest that the strength of existing evidence remains limited and requires further improvement.

### Study limitations

Classification of interventions and heterogeneity: The literature encompasses a variety of exercise modalities, including yoga, resistance training, cycling, and aerobic exercise combined with meditation. The present study did not provide a detailed classification of these intervention types, which may have introduced heterogeneity. The present study did not provide a detailed classification of these intervention types, which may have introduced heterogeneity. Different exercise modalities may involve distinct mechanisms, such as the autonomic regulatory effects of yoga or the neuroendocrine influence of resistance training. Without detailed subgroup analyses, identifying the most effective intervention approach remains difficult, thereby limiting the clinical applicability of the findings. Lack of individualized (precision) interventions: The analysis included limited discussion of exercise intensity and did not tailor interventions to individual characteristics such as body mass index (BMI), lifestyle, or mood phase. For example, patients in the depressive phase may tolerate low-intensity exercise better, whereas those in the manic phase should avoid high-intensity activity that could exacerbate symptoms. Furthermore, the absence of sex-stratified analyses restricts the understanding of sex- or age-related differences in treatment response. Variations in neurodevelopment, hormonal levels, and illness duration may also influence intervention sensitivity (e.g., first-episode vs. chronic patients). The lack of stratified analysis limits the ability to formulate personalized recommendations for specific subgroups. Among the included studies, only two implemented any form of blinding, and most used partial blinding (e.g., assessor blinding only) owing to the practical challenges of fully blinding exercise interventions. This methodological limitation increases the potential for bias. In addition, the majority of studies employed short follow-up periods, preventing evaluation of long-term efficacy—such as sustained mood stabilization or relapse prevention beyond 1 year—and long-term safety outcomes of combined exercise interventions.

### Future research directions

Refinement of intervention classification to reduce heterogeneity: Future research should systematically categorize exercise modalities and clarify the independent effects of different exercise types (e.g., flexibility training, aerobic exercise, or multimodal exercise) when combined with psychological or mindfulness interventions. Subgroup meta-analyses should compare the relative effectiveness of each intervention in improving emotional symptoms, cognitive function, and social functioning, thereby identifying the most effective modality for patients with bipolar disorder. Advancement of precision-based exercise intervention design: Stratification by individual characteristics: Future studies should develop differentiated intervention protocols tailored to patient characteristics such as sex, BMI, mood phase (depressive, manic, or remission), and duration of illness. For example, low-intensity aerobic exercise (e.g., brisk walking) combined with mindfulness breathing may be appropriate for depressive-phase patients with low BMI, whereas low-intensity, low-frequency yoga or meditation programs may be preferable for manic-phase patients to avoid symptom exacerbation. Quantification of intervention intensity: Exercise intensity should be standardized using physiological indicators such as maximum heart rate or metabolic equivalents (METs) to ensure cross-study comparability and to establish measurable criteria for developing clinical exercise prescriptions. Implementation of high-quality study designs and long-term follow-up: Future investigations should prioritize rigorously designed RCTs featuring preregistration and full blinding when feasible—for example, employing “exercise placebo” controls such as light stretching vs. active exercise—to minimize selection, performance, and measurement bias. Expanding sample sizes will increase statistical power, particularly in subgroups undergoing interventions lasting 12 weeks or longer with two to three sessions per week. Moreover, extended follow-up periods of at least 1 year are recommended to assess long-term outcomes such as mood relapse rates, social and academic functioning, interpersonal relationships, and quality of life, thereby providing stronger evidence for sustainable clinical application.

In conclusion, the recommendation for combined exercise and psychological or mindfulness interventions lasting 12 weeks or longer requires further validation through higher-quality evidence. Future research should focus on refining intervention classifications, advancing precision-based designs, and conducting large-scale, high-quality RCTs with long-term follow-up to strengthen the evidence base and provide reliable, evidence-driven guidance for the clinical management of patients with bipolar disorder.

## Data Availability

The datasets presented in this study can be found in online repositories. The names of the repository/repositories and accession number(s) can be found in the article/supplementary material.
