# Supplementary material for: Meta-analysis and systematic review of the impact of different exercise intervention on emotional symptoms in patients with bipolar affective disorders
Source: Front Psychol. 2026 Jan 27;16:1706043. doi: 10.3389/fpsyg.2025.1706043 (PMC12888045; doi:10.3389/fpsyg.2025.1706043)
Supplement: Supplementary file 1 [file Table_1.DOCX]

Appendix

[Appendix1：PRISMA table 2](#_Toc2083)

[Appendix2：PRISMA table 4](#_Toc28005)

[Appendix3：Evidence Quality Evaluation Chart 9](#_Toc3932)

[Appendix4：Search strategy 11](#_Toc13997)

[Appendix5：Publication bias test 14](#_Toc20935)

[Figure 1: Egger's test for depression outcome indicator 14](#_Toc25412)

[Figure 2: Begg's test for depression outcome indicators 15](#_Toc25731)

[Figure 3: Egger's test test for anxiety outcome indicators 16](#_Toc25055)

[Figure 4: Begg Anxiety Outcome Indicator Test 18](#_Toc25553)

[Figure 5: Egger's test for manic outcome indicators 18](#_Toc23771)

[Figure 6: Begg mania result index test 19](#_Toc10002)

[Figure 7: Egger's test for health questionnaire outcome indicators 20](#_Toc13467)

[Figure 8: Begg Health Questionnaire Results Indicator Test 21](#_Toc25312)

[Figure 9: Egger's test for the outcome indicators of the life questionnaire 22](#_Toc16675)

[Figure 10: Begg Life Questionnaire Results Indicator Test 23](#_Toc25111)

**Appendix1：PRISMA table**


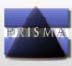
**PRISMA 2020 for Abstracts Checklist**

| **Section and Topic** | **Item #** | **Checklist item** | **Reported (Yes/No)** |
| --- | --- | --- | --- |
| **TITLE** | | |  |
| Title | 1 | Identify the report as a systematic review. | Yes |
| **BACKGROUND** | | |  |
| Objectives | 2 | Provide an explicit statement of the main objective(s) or question(s) the review addresses. | Yes |
| **METHODS** | | |  |
| Eligibility criteria | 3 | Specify the inclusion and exclusion criteria for the review. | Yes |
| Information sources | 4 | Specify the information sources (e.g. databases, registers) used to identify studies and the date when each was last searched. | Yes |
| Risk of bias | 5 | Specify the methods used to assess risk of bias in the included studies. | Yes |
| Synthesis of results | 6 | Specify the methods used to present and synthesise results. | Yes |
| **RESULTS** | | |  |
| Included studies | 7 | Give the total number of included studies and participants and summarise relevant characteristics of studies. | Yes |
| Synthesis of results | 8 | Present results for main outcomes, preferably indicating the number of included studies and participants for each. If meta-analysis was done, report the summary estimate and confidence/credible interval. If comparing groups, indicate the direction of the effect (i.e. which group is favoured). | Yes |
| **DISCUSSION** | | |  |
| Limitations of evidence | 9 | Provide a brief summary of the limitations of the evidence included in the review (e.g. study risk of bias, inconsistency and imprecision). | Yes |
| Interpretation | 10 | Provide a general interpretation of the results and important implications. | Yes |
| **OTHER** | | |  |
| Funding | 11 | Specify the primary source of funding for the review. | Yes |
| Registration | 12 | Provide the register name and registration number. | Yes |

*From:*  Page MJ, McKenzie JE, Bossuyt PM, Boutron I, Hoffmann TC, Mulrow CD, et al. The PRISMA 2020 statement: an updated guideline for reporting systematic reviews. BMJ 2021;372:n71. doi: 10.1136/bmj.n71

For more information, visit: <http://www.prisma-statement.org/>

**Appendix2：PRISMA table**

Appendix S1 – PRISMA 2020 statement


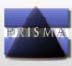


| **Section and Topic** | **Item #** | **Checklist item** | **Location where item is reported** |
| --- | --- | --- | --- |
| **TITLE** | | |  |
| Title | 1 | Identify the report as a systematic review. | Title page |
| **ABSTRACT** | | |  |
| Abstract | 2 | See the PRISMA 2020 for Abstracts checklist. | Abstracts checklist |
| **INTRODUCTION** | | |  |
| Rationale | 3 | Describe the rationale for the review in the context of existing knowledge. | Page 2-3 |
| Objectives | 4 | Provide an explicit statement of the objective(s) or question(s) the review addresses. | Page 3 |
| **METHODS** | | |  |
| Eligibility criteria | 5 | Specify the inclusion and exclusion criteria for the review and how studies were grouped for the syntheses. | Page 4 |
| Information sources | 6 | Specify all databases, registers, websites, organisations, reference lists and other sources searched or consulted to identify studies. Specify the date when each source was last searched or consulted. | Page 4 |
| Search strategy | 7 | Present the full search strategies for all databases, registers and websites, including any filters and limits used. | Page 4 & Appendix 4 |
| Selection process | 8 | Specify the methods used to decide whether a study met the inclusion criteria of the review, including how many reviewers screened each record and each report retrieved, whether they worked independently, and if applicable, details of automation tools used in the process. | Page 4 |
| Data collection process | 9 | Specify the methods used to collect data from reports, including how many reviewers collected data from each report, whether they worked independently, any processes for obtaining or confirming data from study investigators, and if applicable, details of automation tools used in the process. | Page 4 |
| Data items | 10a | List and define all outcomes for which data were sought. Specify whether all results that were compatible with each outcome domain in each study were sought (e.g. for all measures, time points, analyses), and if not, the methods used to decide which results to collect. | Page 4 |
|  | 10b | List and define all other variables for which data were sought (e.g. participant and intervention characteristics, funding sources). Describe any assumptions made about any missing or unclear information. |  |
| Study risk of bias assessment | 11 | Specify the methods used to assess risk of bias in the included studies, including details of the tool(s) used, how many reviewers assessed each study and whether they worked independently, and if applicable, details of automation tools used in the process. | Page 5 |
| Effect measures | 12 | Specify for each outcome the effect measure(s) (e.g. risk ratio, mean difference) used in the synthesis or presentation of results. | Page 5 |
| Synthesis methods | 13a | Describe the processes used to decide which studies were eligible for each synthesis (e.g. tabulating the study intervention characteristics and comparing against the planned groups for each synthesis (item #5)). | Page 5 |
|  | 13b | Describe any methods required to prepare the data for presentation or synthesis, such as handling of missing summary statistics, or data conversions. |  |
|  | 13c | Describe any methods used to tabulate or visually display results of individual studies and syntheses. |  |
|  | 13d | Describe any methods used to synthesize results and provide a rationale for the choice(s). If meta-analysis was performed, describe the model(s), method(s) to identify the presence and extent of statistical heterogeneity, and software package(s) used. | Page 5 |
|  | 13e | Describe any methods used to explore possible causes of heterogeneity among study results (e.g. subgroup analysis, meta-regression). | Page 5 |
|  | 13f | Describe any sensitivity analyses conducted to assess robustness of the synthesized results. | Page 5 |
| Reporting bias assessment | 14 | Describe any methods used to assess risk of bias due to missing results in a synthesis (arising from reporting biases). | Page 5 |
| Certainty assessment | 15 | Describe any methods used to assess certainty (or confidence) in the body of evidence for an outcome. | Page 5 |
| **RESULTS** | | |  |
| Study selection | 16a | Describe the results of the search and selection process, from the number of records identified in the search to the number of studies included in the review, ideally using a flow diagram. | Page 6 & Fig. 1 |
|  | 16b | Cite studies that might appear to meet the inclusion criteria, but which were excluded, and explain why they were excluded. | Page 6 & Fig. 1 |
| Study characteristics | 17 | Cite each included study and present its characteristics. | Page 6 & Table 1 |
| Risk of bias in studies | 18 | Present assessments of risk of bias for each included study. | Page 6 & Fig. 2 |
| Results of individual studies | 19 | For all outcomes, present, for each study: (a) summary statistics for each group (where appropriate) and (b) an effect estimate and its precision (e.g. confidence/credible interval), ideally using structured tables or plots. | Page . 6-8 & Fig3-9 |
| Results of syntheses | 20a | For each synthesis, briefly summarise the characteristics and risk of bias among contributing studies. | Page 6-8 |
|  | 20b | Present results of all statistical syntheses conducted. If meta-analysis was done, present for each the summary estimate and its precision (e.g. confidence/credible interval) and measures of statistical heterogeneity. If comparing groups, describe the direction of the effect. | Page 6-8 |
|  | 20c | Present results of all investigations of possible causes of heterogeneity among study results. | Page 8-10，Table 2 Table 3 |
|  | 20d | Present results of all sensitivity analyses conducted to assess the robustness of the synthesized results. | Page 10 & Fig. 10-16 |
| Reporting biases | 21 | Present assessments of risk of bias due to missing results (arising from reporting biases) for each synthesis assessed. | Page 10-11 & Table 4 Table 5 Fig. 17-23. Appendix 5 |
| Certainty of evidence | 22 | Present assessments of certainty (or confidence) in the body of evidence for each outcome assessed. | Page 11 & Appendix 3 |
| **DISCUSSION** | | |  |
| Discussion | 23a | Provide a general interpretation of the results in the context of other evidence. | Page 11 & Appendix 3 |
|  | 23b | Discuss any limitations of the evidence included in the review. | Page 11 |
|  | 23c | Discuss any limitations of the review processes used. | Page 11-15 |
|  | 23d | Discuss implications of the results for practice, policy, and future research. | Page 15 |
| **OTHER INFORMATION** | | |  |
| Registration and protocol | 24a | Provide registration information for the review, including register name and registration number, or state that the review was not registered. | Title page |
|  | 24b | Indicate where the review protocol can be accessed, or state that a protocol was not prepared. |  |
|  | 24c | Describe and explain any amendments to information provided at registration or in the protocol. |  |
| Support | 25 | Describe sources of financial or non-financial support for the review, and the role of the funders or sponsors in the review. | Page 15 |
| Competing interests | 26 | Declare any competing interests of review authors. | Title page |
| Availability of data, code and other materials | 27 | Report which of the following are publicly available and where they can be found: template data collection forms; data extracted from included studies; data used for all analyses; analytic code; any other materials used in the review. |  |

*From:*  Page MJ, McKenzie JE, Bossuyt PM, Boutron I, Hoffmann TC, Mulrow CD, et al. The PRISMA 2020 statement: an updated guideline for reporting systematic reviews. BMJ 2021;372:n71. doi: 10.1136/bmj.n71

For more information, visit: <http://www.prisma-statement.org/>

**Appendix3：Evidence Quality Evaluation Chart**


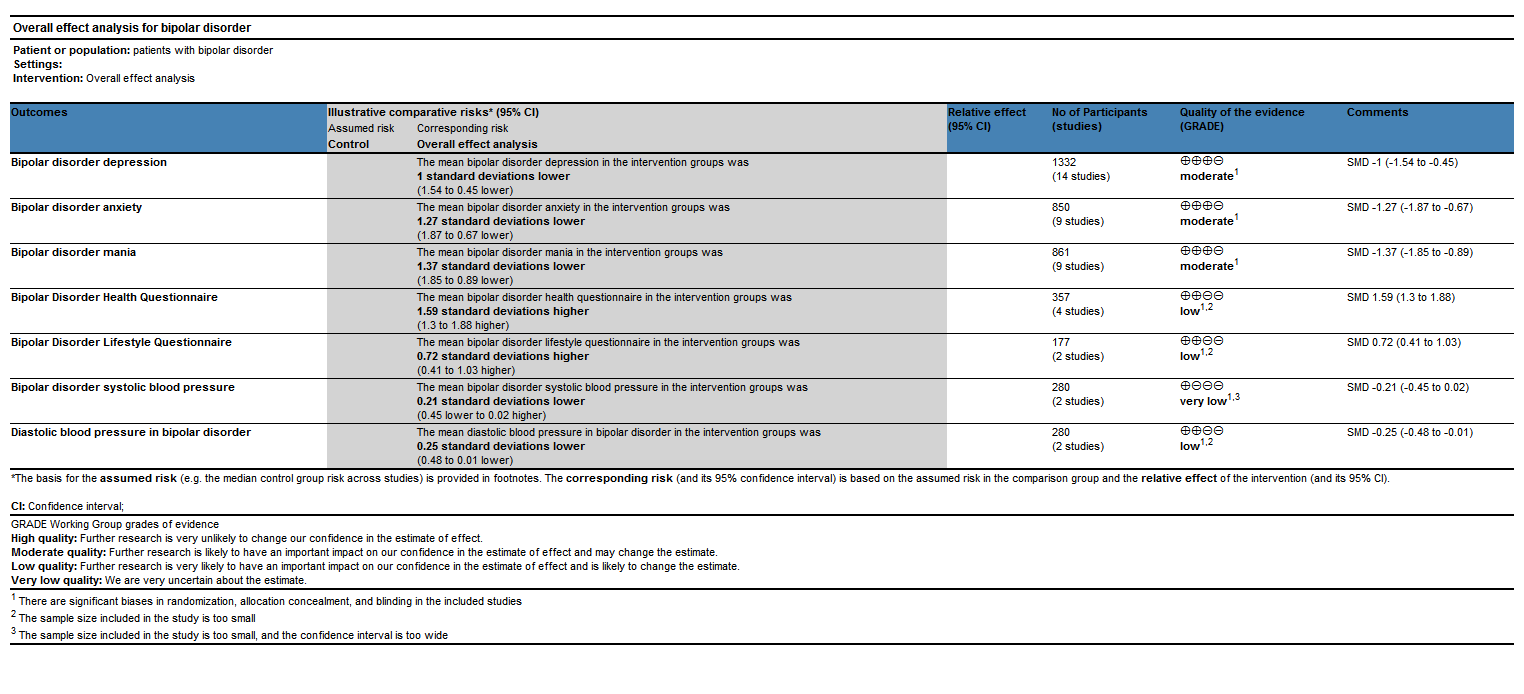


**Appendix4：Search strategy**

**[Cochrane library](https://www.bing.com/ck/a?!&&p=13d69e51766fb78bJmltdHM9MTcxMDExNTIwMCZpZ3VpZD0wZWYzMmU0ZS1iODY0LTYzOGYtMzNiYi0zZGEwYjk0YTYyY2UmaW5zaWQ9NTU0Mw&ptn=3&ver=2&hsh=3&fclid=0ef32e4e-b864-638f-33bb-3da0b94a62ce&u=a1L3NlYXJjaD9xPWNvY2hyYW5lK2xpYnJhcnklZTUlYWUlOTglZTclYmQlOTElZTUlODUlYTUlZTUlOGYlYTMmRk9STT1RU1JFMQ&ntb=1)**

#1sport

#2(sport):ti,ab,kw OR (exercise):ti,ab,kw OR (physical activity):ti,ab,kw OR (training):ti,ab,kw OR (yoga):ti,ab,kw

#3(shadow boxing):ti,ab,kw OR (traditional sports items):ti,ab,kw

#4：#1or#2or#3

#5：bipolar depression

#6(bipolar depression):ti,ab,kw OR (Type 2 Bipolar Disorder):ti,ab,kw OR (Psychoses, Manic Depressive):ti,ab,kw OR (Bipolar Affective Psychosis):ti,ab,kw OR (Manic-Depressive Psychosis):ti,ab,kw

#7：(Bipolar Mood Disorder):ti,ab,kw OR (Bipolar Disorder Type 1):ti,ab,kw

#8：#5or#6or#7

#9：Randomized controlled trial

#10：#4and#8and#9

**PubMed**

**#1：Search:((((((sport[Title/Abstract]) OR (exercise[Title/Abstract])) OR (training[Title/Abstract])) OR (physical activity[Title/Abstract])) OR (yoga[Title/Abstract])) OR (shadow boxing[Title/Abstract])) OR (traditional sports items[Title/Abstract])**

**#2：Search: (((((((((((Bipolar Disorders[Title/Abstract]) OR (Disorder, Bipolar[Title/Abstract])) OR (Bipolar Mood Disorder[Title/Abstract])) OR (Manic Depression[Title/Abstract])) OR (Depression, Manic[Title/Abstract])) OR (Affective Psychosis, Bipolar[Title/Abstract])) OR (Bipolar Affective Psychosis[Title/Abstract])) OR (Psychoses, Bipolar Affective[Title/Abstract])) OR (Manic-Depressive Psychosis[Title/Abstract])) OR (Bipolar Disorder Type 1[Title/Abstract])) OR (Major Affective Disorder 2[Title/Abstract])) OR (Manic Disorders[Title/Abstract])**

**#3： Randomized controlled trial[Title/Abstract]**

**#4：Search: ((Randomized controlled trial[Title/Abstract]) AND ((((((((((((Bipolar Disorders[Title/Abstract]) OR (Disorder, Bipolar[Title/Abstract])) OR (Bipolar Mood Disorder[Title/Abstract])) OR (Manic Depression[Title/Abstract])) OR (Depression, Manic[Title/Abstract])) OR (Affective Psychosis, Bipolar[Title/Abstract])) OR (Bipolar Affective Psychosis[Title/Abstract])) OR (Psychoses, Bipolar Affective[Title/Abstract])) OR (Manic-Depressive Psychosis[Title/Abstract])) OR (Bipolar Disorder Type 1[Title/Abstract])) OR (Major Affective Disorder 2[Title/Abstract])) OR (Manic Disorders[Title/Abstract]))) AND (((((((sport[Title/Abstract]) OR (exercise[Title/Abstract])) OR (training[Title/Abstract])) OR (physical activity[Title/Abstract])) OR (yoga[Title/Abstract])) OR (shadow boxing[Title/Abstract])) OR (traditional sports items[Title/Abstract]))**

**Embase**

#1：(sport:ab,ti OR exercise:ab,ti OR 'physical activity':ab,ti OR training:ab,ti OR yoga:ab,ti OR 'shadow boxing':ab,ti OR 'traditional sports items':ab,ti)

#2：（bipolar disorder':ab,ti OR 'bipolar disorders':ab,ti OR 'manic depression':ab,ti OR 'treatment resistant bipolar depression':ab,ti OR 'bipolar disorder type 1':ti OR 'bipolar disorder type 2':ab,ti OR 'major affective disorder 2':ab,ti OR 'depression, manic':ab,ti）

#3：（randomized controlled tria':ab,ti）

#4：#1and#2and#3

**Scopus**

#1:( TITLE-ABS-KEY ( sport ) OR TITLE-ABS-KEY ( exercise ) OR TITLE-ABS-KEY ( physical AND activity ) OR TITLE-ABS-KEY ( training ) OR TITLE-ABS-KEY ( yoga ) OR TITLE-ABS-KEY ( shadow AND boxing ) OR TITLE-ABS-KEY ( traditional AND sports AND items ) )
#2:( TITLE-ABS-KEY ( bipolar AND disorder ) OR TITLE-ABS-KEY ( bipolar AND disorders ) OR TITLE-ABS-KEY ( manic AND depression ) OR TITLE-ABS-KEY ( bipolar AND disorder AND type 1 ) OR TITLE-ABS-KEY ( bipolar AND disorder AND type 2 ) OR TITLE-ABS-KEY ( depression, AND manic ) OR TITLE-ABS-KEY ( bipolar AND depression ) )

#3:TITLE-ABS-KEY ( randomized AND controlled AND trial )

#4:( ( TITLE-ABS-KEY ( sport ) OR TITLE-ABS-KEY ( exercise ) OR TITLE-ABS-KEY ( physical AND activity ) OR TITLE-ABS-KEY ( training ) OR TITLE-ABS-KEY ( yoga ) OR TITLE-ABS-KEY ( shadow AND boxing ) OR TITLE-ABS-KEY ( traditional AND sports AND items ) ) ) AND ( ( TITLE-ABS-KEY ( bipolar AND disorder ) OR TITLE-ABS-KEY ( bipolar AND disorders ) OR TITLE-ABS-KEY ( manic AND depression ) OR TITLE-ABS-KEY ( bipolar AND disorder AND type 1 ) OR TITLE-ABS-KEY ( bipolar AND disorder AND type 2 ) OR TITLE-ABS-KEY ( depression, AND manic ) OR TITLE-ABS-KEY ( bipolar AND depression ) ) ) AND ( TITLE-ABS-KEY ( randomized AND controlled AND trial ) )

**Wos**

#1：((((((TS=(sport)) OR TS=(exercise)) OR TS=(physical activity)) OR TS=(training)) OR TS=(Yoga )) OR TS=(shadow boxing)) OR TS=(traditional sports items) and Preprint Citation Index (Exclude – Database)

#2：(((((((TS=(bipolar disorder)) OR TS=(bipolar disorders)) OR TS=(manic depression)) OR TS=(bipolar disorder type 1)) OR TS=(bipolar disorder type 2)) OR TS=(major affective disorder 2)) OR TS=(depression, manic)) OR TS=(Bipolar Depression) and Preprint Citation Index (Exclude – Database)

#3：TS=(Randomized controlled trial) and Preprint Citation Index (Exclude – Database)

#4：#1 AND #2 AND #3 and Preprint Citation Index (Exclude – Database)

**CNKI**
TKA=(训练 + 身体活动 + 运动 + 体育锻炼 + 瑜伽 + 太极拳 + 传统体育项目) AND TKA=(双相情感障碍 + 重度抑郁 + 狂躁 + 狂躁症 + 情感障碍 ) 528

**Weipu**

(U=(运动 or 锻炼 or 身体活动 or 体育训练 or 瑜伽 or 太极拳 or 传统体育项目 )) AND (U=(双相情感障碍 or 情感障碍 or 情绪障碍 or 重度抑郁 or 狂躁 or 狂躁症))166

**Wanfang**

(("运动" or "锻炼" or "身体活动" or "体育训练" or"瑜伽"or"太极拳"or"传统体育项目" ) and and ("双相情感障碍" or "情感障碍"or"情绪障碍"or"重度抑郁"or"狂躁"or"狂躁症")) 594

**Sinomed**

#1： "运动"[常用字段:智能] OR "锻炼"[常用字段:智能] OR "身体活动"[常用字段:智能] OR "体育训练"[常用字段:智能] OR "瑜伽"[常用字段:智能] OR "太极拳"[常用字段:智能] OR "传统体育项目"[常用字段:智能]

#2： "双相情感障碍"[常用字段:智能] OR "情感障碍"[常用字段:智能] OR "狂躁症"[常用字段:智能] OR "狂躁"[常用字段:智能] OR "重度抑郁"[常用字段:智能] OR "情绪障碍"[常用字段:智能]

#3： "运动"[常用字段:智能] OR "锻炼"[常用字段:智能] OR "身体活动"[常用字段:智能] OR "体育训练"[常用字段:智能] OR "瑜伽"[常用字段:智能] OR "太极拳"[常用字段:智能] OR "传统体育项目"[常用字段:智能]and "运动"[常用字段:智能] OR "锻炼"[常用字段:智能] OR "身体活动"[常用字段:智能] OR "体育训练"[常用字段:智能] OR "瑜伽"[常用字段:智能] OR "太极拳"[常用字段:智能] OR "传统体育项目"[常用字段:智能]

**Appendix5：Publication bias test**

Figure 1: Egger's test for depression outcome indicator

Figure 2: Begg's test for depression outcome indicators

Figure 3: Egger's test test for anxiety outcome indicators

Figure 4: Begg Anxiety Outcome Indicator Test

Figure 5: Egger's test for manic outcome indicators

Figure 6: Begg mania result index test

Figure 7: Egger's test for health questionnaire outcome indicators

Figure 8: Begg Health Questionnaire Results Indicator Test

Figure 9: Egger's test for the outcome indicators of the life questionnaire

Figure 10: Begg Life Questionnaire Results Indicator Test

Figure 11: Egger's test plot for the outcome indicator of systolic blood pressure

Figure 12: Begg's systolic blood pressure result index test

Figure 13: Egger's test plot for diastolic blood pressure outcome indicator

Figure 14: Begg's diastolic blood pressure result index test
